# Opportunities for RNA sequencing in physiology: from big data to understanding homeostasis and heterogeneity

**DOI:** 10.1152/function.019.2025

**Published:** 2025-12-05

**Authors:** Jeremy W. Prokop, Stephanie M. Bilinovich, Ember Tokarski, Sangeetha Vishweswaraiah, Sophie VanderWeele, Akansha S. Das, Surya B. Chhetri, Alexander Dao, Sanjana Arora, Austin Goodyke, Katie L. Buelow, Mason Westgate, Elizabeth A. VanSickle, Claudia J. Edell, Lance N. Benson, Daniel B. Campbell, Caleb P. Bupp, Amanda Holsworth, Nicholas L. Hartog, Jena M. Krueger, Marcos Cordoba, Matthew Sims, Maximiliano A. Tamae Kakazu, Angela M. Peraino, Stewart F. Graham, Tim Triche, Elora Hussain, Mara L. Leimanis-Laurens, Connie M. Krawczyk, Jennifer S. Pollock, Surender Rajasekaran

**Affiliations:** ^1^Corewell Health Research Institute, Corewell Health, Grand Rapids, Michigan, United States; ^2^College of Human Medicine, Michigan State University, Grand Rapids, Michigan, United States; ^3^Department of Chemistry, Grand Valley State University, Allendale, Michigan, United States; ^4^Center for Epigenetics, Van Andel Institute, Grand Rapids, Michigan, United States; ^5^Department of Obstetrics and Gynecology, Oakland University William Beaumont School of Medicine, Royal Oak, Michigan, United States; ^6^Department of Biological Engineering, Massachusetts Institute of Technology, Cambridge, Massachusetts, United States; ^7^Center for Computational Biology, Johns Hopkins University, Baltimore, Maryland, United States; ^8^Section of Cardio-Renal Physiology and Medicine, Division of Nephrology, Department of Medicine, University of Alabama at Birmingham, Birmingham, Alabama, United States; ^9^Medical Genetics, Corewell Health, Grand Rapids, Michigan, United States; ^10^Allergy and Immunology, Corewell Health, Grand Rapids, Michigan, United States; ^11^Department of Neurology, Helen DeVos Children's Hospital, Corewell Health, Grand Rapids, Michigan, United States; ^12^Department of Obstetrics, Gynecology and Women's Health, Corewell Health, Grand Rapids, Michigan, United States; ^13^Section of Infectious Diseases and International Medicine, Department of Internal Medicine, Beaumont Royal Oak, Royal Oak, Michigan, United States; ^14^Division of Pulmonary and Critical Care, Corewell Health, Grand Rapids, Michigan, United States; ^15^Pediatric Intensive Care Unit, Helen DeVos Children's Hospital, Corewell Health, Grand Rapids, Michigan, United States; ^16^Department of Metabolism and Nutritional Programming, Van Andel Institute, Grand Rapids, Michigan, United States

**Keywords:** heterogeneous response, homeostasis, physiological omics, single-cell RNA sequencing, spatial transcriptomics

## Abstract

The quantity of physiological data has grown exponentially, yielding insights into mechanisms of phenotypic and disease pathways. Among the powerful tools for physiological omics is the study of RNA, where broad sequencing of RNA leads to hypothesis generation and testing while providing observational discovery. Emphasis has been placed on RNA molecules that code for proteins, even though they represent a minority of total RNA. Diverse sequencing methods have rapidly expanded the identification of non-protein-coding molecules, including nonsense-mediated decay and long non-coding RNAs (lncRNA), which now represent the most diverse class of RNA. Increasing attention needs to be paid to the data processing of RNA sequencing to interpret transcript-level mapping data in the context of protein biology, as many protein-coding genes have diverse noncoding transcripts. Over the past several years, single-cell and spatial transcriptomics have yielded unprecedented insights into cellular, tissue, and organ physiology. Building on these advancements, bulk RNA sequencing tools have begun producing robust deconvolution methods that enhance the analysis of human genes, the detection of foreign RNA from bacteria and viruses, and provide deep insights into complex immunological events, such as B- and T-cell recombination. Over a million RNA-sequencing datasets have been generated, providing resources for data scientists to reprocess data and expand larger databases. From model organisms to complex human diseases, RNA sequencing resources continue to transform our knowledge of the complexity of personalized disease insights. Observational science is at the core of physiology, and growth of RNA sequencing represents a significant tool for physiologists.

## Graphical abstract

**Figure:**
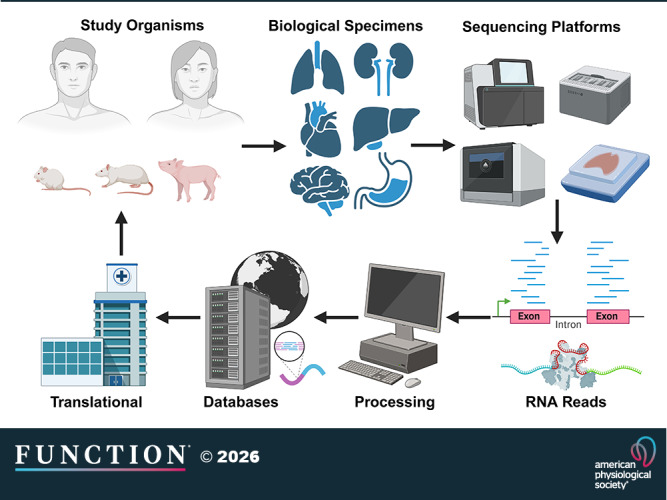

## INTRODUCTION

The Greek word physiologos translates to “the study of nature,” suggesting that a physiologist is an observer who interprets. Physiology, the science of how living things function, was born from observational sciences. Hippocrates’ studies of the body’s humors that form the foundations of homeostasis and the search for cause and effect led to an appreciation for the complex systems within living species.

Stemming from the growth of anatomical knowledge and investigations into diseases, insights elevated through observation of biological systems, giving rise to testable hypotheses and model organism research that became experimental physiology (1–3). The observations were powered by advanced tools of recording instruments (blood pressure, heart rate, electrophysiology, and lung capacity) that enabled measurements to be processed for statistical power (4). Similar to the early days of physiology, expanding tools such as DNA and RNA sequencing, along with other omics, enabled a new age of observational science that significantly enhanced our ability to form unbiased hypotheses. With the launch of the journal *Physiological Genomics* in 1999 by the American Physiological Society, a new era of omic-centric physiology was born (5).

Of the RNA within a cell, 98% of transcriptional output is noncoding and represents many undefined physiological mechanisms in cell regulation, structural biology, RNA interference, cosuppression, transgene silencing, imprinting, and methylation (6). Although the human genome codes for 19,433 protein-coding genes, there are 507,365 uniquely spliced RNA sequences from 78,691 unique genes known as of 2025 and 278,326 transcripts from 78,275 genes in mice (7), representing a significant volume of underexplored biology. These numbers do not account for the unique splicing that can result in individuals due to genetic variants, the complex genome rearrangements within the acquired immune system, and the millions of transcripts from species found within the biomass of humans (8). Although multiple genes have been extensively established for involvement within physiology, many of the human and mouse transcripts remain understudied, with the majority of transcripts with one or fewer publications addressing their biology, especially those RNA molecules that do not code for a protein (9).

Two hallmark concepts taught in physiology are homeostasis and heterogeneity (10–12). The emerging tools of RNA sequencing continue to advance our knowledge of both concepts, where the tools enable broad mapping while giving fine details to develop testable hypotheses at the cellular and spatial levels (8). The emergence of precision medicine, which can pair phenotypes with genomics, is potentiated by RNA sequencing and other omics to address clinical insights, which has expanded our knowledge of homeostasis and heterogeneity.

RNA homeostasis coordinates volume, microRNAs, cell fate, and senescence at the cellular level through gene regulation and RNA turnover (13–15). These studies are being increasingly powered by advancing tools of single-cell and spatial RNA sequencing (16–18). Imbalance of RNA homeostasis has been suggested in states such as cancer (19), inflammatory (20), stress (21), aging (22), and cardiovascular disease (23, 24), to name a few. A central aspect of physiology is the integration of many phenotypes into simple logistical mechanisms, where RNA sequencing elevates our insights and connects diverse phenotypes through homeostatic circuits.

Human genome diversity has gained an appreciation for modifying disease pathology, where genome sequencing tools have enabled these studies (25). Within areas such as cystic fibrosis, the same genetic variant can manifest with highly diverse phenotypes, where factors from genetic modifiers to infection status can modulate nearly every disease factor (26). This has given rise to fields such as response genetics, where scientists can study complex genetics by environmental factor interactions (27–29). These environmental factors are not addressed by genome sequencing alone but require additional omic metrics. In this review article, we will highlight how RNA sequencing can embrace both homeostasis and heterogeneity.

As the cost of these sequencing tools has decreased, it has opened the door to the idea that simple hypothesis testing with RNA sequencing outperforms single RNA molecule tracking with real-time PCR. This has enabled hypothesis testing to be paired with broader, unbiased observational sciences that can reframe scientific discovery if proper analysis is given. As the number of tools for RNA sequencing has grown, it has unfortunately also created challenges in identifying the right tool for the right project, as some of the tools have higher expenses that limit their broader scaling, yet they provide cellular or spatial resolution of knowledge. As most sequencing machines have a high price tag, this has led to the concentration on sequencing centers and cores, facilitating higher volumes and lower costs. Although this has been beneficial in bringing these tools into more laboratories with a lower entry point, it has also decreased the number of experiments where the sequencing chemistry itself is variable, thereby producing less variability in types of discoveries. Labs often rely on one method over others for the simplicity of working with cores, reducing the options for optimizing the right tools for the right questions.

Within this review, we discuss the current state of tools for RNA sequencing, examples of usages within physiology, and the areas that are likely to continue powering physiologists. The article aims to provide a primer to scientists and trainees new to the field while guiding future work into rapidly emerging areas. The goal is to provide a community resource to match the RNA sequencing tools to projects and assist the community in advancing the field of physiology.

## THE HUMAN GENOME TRANSITION TO TRANSCRIPT DISCOVERY

The pilot human genome, completed in 2003–2004, enabled the new era of sequencing-based discoveries, with 99% of the genome mapped, enabling searching for RNA coding genes (30). The genome was narrowed to approximately 20,000–25,000 protein-coding genes based on bioinformatic filtering of open reading frames. The sequencing of genomes was powered by next-generation sequencing, DNA fragmentation, and DNA sequencing by polymerase fluorescent reactions imaged on high-resolution cameras (31, 32).

With the advancements of the next-generation platforms, researchers paired cDNA synthesis from RNA as input into the sequencing to map the coded RNA from specimens (33). Taking all RNA and making cDNA results in most sequencing for ribosomal RNA (rRNA), given that it represents approximately 80% of RNA volume within human cells. Researchers primarily focused on oligo-dT-based capture to remove the rRNA and capture the polyadenylation (polyA)-enriched protein-coding messenger RNA (mRNA) class (34). The ENCyclopedia Of DNA Elements (ENCODE) (35) and GENCODE initiatives (36), launched shortly after the draft genome completion, organized the mapping of RNA molecules and built gene regulation insights. In 2005–2006, the initial Gencode release started to develop maps of genes (37). By 2012, the GENCODE release mapped 20,687 protein-coding and 9,640 long non-coding RNA (lncRNA) loci (36).

The early RNA sequencing studies were focused on which DNA elements were transcribed into RNA (37), the mapping of transcriptional start sites within the genome based on 5′ untranslated region (UTR) extensions of RNA (38), how those RNAs get spliced (39), and how RNA molecules can be fused within cancer (40). Further techniques to remove the rRNA through degradation (ribo-reduced) enabled the cDNA conversion of a broader class of RNA molecules (41) while also capturing bacterial RNA that is not polyA-modified (42). By 2021, the GENCODE reference mapped protein-coding genes shrunk to 19,954, while the lncRNA scaled to 17,957 loci (43).

The original next-generation sequencing technologies were limited in understanding splicing complexity due to the short-read chemistry, where fragments are sequenced for 50–300 bases at a time. Thus, connecting splicing sites more than a few hundred bases apart was challenging to perform through bioinformatic technologies. Advancing technologies, such as single molecular PacBio and protein pore-based nanopore, were able to generate long reads of RNA that vastly enhanced our knowledge of splicing and non-coding RNA (44, 45). By 2025, the GENCODE map showed stability of protein-coding genes (19,433) while there has been a massive amplification of known non-coding RNA (lncRNA 35,899) (46).

As the transcript and gene maps were refined, additional methods to capture RNA were developed. These included capturing RNA within specific compartments such as the nucleus (47–49) or bound to ribosomes in active translation (50–52). Cells could be sorted through various platforms, and the RNA barcoded to open the door for single-cell RNA sequencing (53–55). Histology sections could be placed on barcoded surfaces, imaged, and sequenced to develop spatial transcriptomics (56–58). More recently, technological advances in nanopore chemistries have removed the need for cDNA conversion, with the possibility of directly sequencing RNA molecules that include hundreds of chemical RNA-base modifications (59, 60). Often overlooked for the advancement of RNA sequencing are the incredible commercial advancements in RNA stabilization reagents, novel collection methods, and RNA purification systems that have powered the collections (61). As the past two decades have highlighted, the technological advancements for RNA sequencing have refined our knowledge of the transcript landscape and enabled powerful tools for physiological characterizations.

## THE HUMAN AND MOUSE TRANSCRIPTOMES

**Figure 1.**
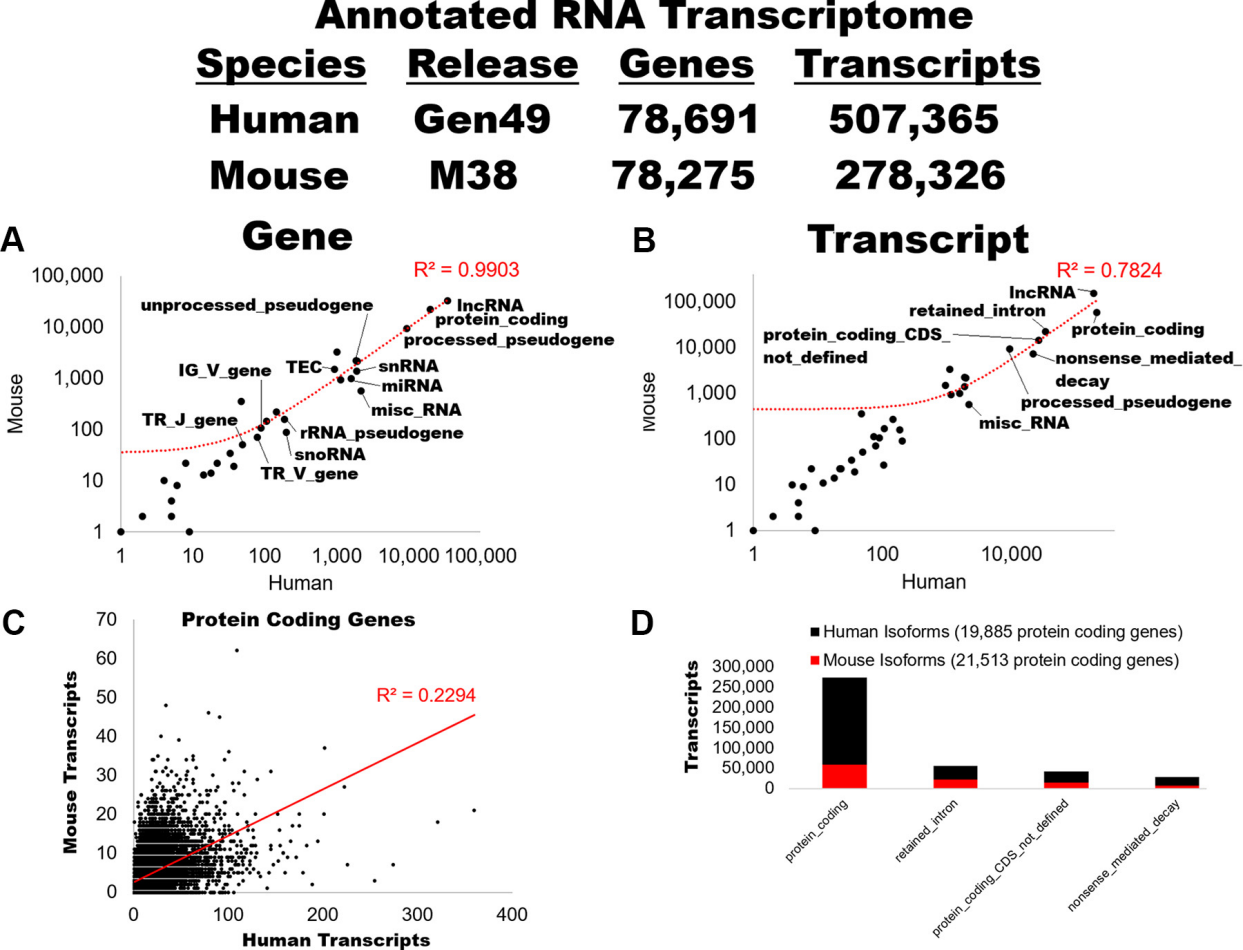
Current knowledge of human and mouse RNA biotypes. *A* and *B*: the number of each biotype in the reference Gencode transcripts for human (Gen49, *x*-axis) and mouse (M38, *y*-axis) for genes (*A*) and transcripts (*B*). *C*: number of transcripts per protein coding gene for humans (*x*-axis) and mice (*y*-axis). *D*: the number of transcripts of diverse biotypes for genes with human and mice protein-coding transcripts.

The 49th GENCODE version of the human and 38th mouse transcriptomes were released in September of 2025 (Fig. 1) (46). The human release contained 78,691 total genes with 507,365 known spliced transcripts. The mouse release contained 78,275 total genes with 278,326 known spliced transcripts. This release showed the most considerable growth of non-coding RNA transcripts of any previous GENCODE version, representing technological advances in targeted capturing and long-read sequencing platforms. The lower number of transcripts within the mouse likely reflects less utilization of long-read platforms to generate broad tissue data, which have recently been applied to human samples. Based on quasi-alignment filtering (62), we present the transcripts from diverse biotypes for genes that are highly concordant in human and mouse (Fig. 1*A*), whereas transcript biology shows more divergence between the species (Fig. 1*B*). It is worth noting that many transcripts can be classified into multiple biotype categories. We have utilized the Gencode single annotation events for simplicity. In the text below, we do not focus on the well-reviewed protein-coding transcripts, which others have extensively reviewed (8, 63–65).

### Long Noncoding RNA

The largest group of known transcripts comes from lncRNA, representing 45.6%/46.1% (human/mouse) of all genes and 37.7%/56.0% of transcripts. The noncoding RNA molecules are the most diverse of any biotype and represent the largest biomass within cells, where ribosomal RNA accounts for around 80% of the total biomass. A few lncRNA molecules, such as XIST, have been shown to have pivotal roles in processes such as X-chromosome inactivation (XCI; 66). In many RNA sequencing experiments of physiological processes and diseases, lncRNA molecules are differentially expressed (67). Human and model organism biology, including obesity (68), cancer (69), homeostasis (70), and cardiovascular (71), have shown the involvement of lncRNA in phenotypic variability. The advent of CRISPR-based technologies is quickly identifying lncRNA functional mechanisms, such as the recent work linking the lncRNA *KILR* in breast cancer and the regulation of DNA replication/repair processes (72). The physiological processes for lncRNA continue to grow to include gene regulation, structural biology, posttranscriptional control, splicing, and posttranslational modifications (73–75).

Deciphering the molecular function of lncRNA has been challenging due to the lack of molecular biology knowledge and species specificity, to produce limited bioinformatic tools for processing the thousands of lncRNA gene sequences for function (76–78). Many genome or phenome-wide association (GWAS/PheWAS) studies have fine-mapped genetic variants found within lncRNA. Yet, these variants have not been functionally characterized (79, 80). LncRNA likely represents a wide array of mechanisms that have yet to be discovered (81), suggesting an important area for future research within physiology.

### Retained Intron RNA

Retained intron transcripts represent around 6.7% of all known transcripts. These transcripts are often from protein-coding genes that get processed and withhold an intron or part of an intron into the final mature product, resulting in the loss of the open reading frame sequence of the protein, which often results in controlled degradation (82). However, alterations in the degradation pathways of retained intron transcripts can elevate their presence in cells (83). These transcripts can be challenging to detect reliably in sequencing and bioinformatics (84, 85). The biological roles of retained intron transcripts have been growing, where they are critical for neuron differentiation (86), Alzheimer’s disease (87), aging (88), hypoxia (89), activation of CD4 T cells (90), and cardiovascular diseases (91).

### Nonsense-Mediated Decay RNA

Nonsense-mediated decay (NMD) transcripts represent around 3%–5% of all known transcripts and have an early stop codon before the last splicing site of the transcript (92, 93). This results in the accumulation of proteins bound to the RNA that are not removed by ribosomes in translation, resulting in a controlled degradation of the RNA (94–96). NMD transcripts are well-studied in rare diseases. In many cases of splice or nonsense variants resulting in early stop codons, the NMD degradation results in haploinsufficiency of the RNA, where transcript abundance can be compared with the normal allele, resulting in an allelic imbalance in expression (97, 98). In some cases of rare disease splice or nonsense variants, NMD does not degrade the early termination transcript, resulting in a partial protein that can produce autosomal dominant negative phenotypes (99, 100). An example is the recent discovery of a *RABL3* early stop codon that avoids NMD regulation for familial pancreatic ductal adenocarcinoma through a 36 amino acid peptide-based disruption of KRAS biology (101).

Various biological processes, such as development, cancer, infections, stress response, T-cell receptor selection, and aging, are known to alter the NMD degradation processes (102–106). When NMD is inhibited, the protein products of NMD transcripts can result in partial proteins with either de novo function relative to the full-length protein-coding genes or in dominant negative forms of biological competition for the full-length proteins (107). The nature of environmental regulation of NMD produces a rapid sensing switch within cells, where the NMD transcripts are continually made, but their accumulation through NMD inhibition results in rapid cellular changes. Endoplasmic reticulum (ER) stress is a well-studied environmental control mechanism (108). Factors such as elevated protein-folding-derived ER stress have been shown to activate quality control mechanisms that include the suppression of NMD degradation and altered transcripts of genes such as *UPF1* (109, 110).

The balance of preventing deleterious early stop codons from impacting physiology in rare diseases and the environmental sensing mechanisms makes NMD transcripts important for future research (111). Advancing treatments, including exon skipping and splice site-altering oligonucleotides, hold incredible promise for NMD-based genetic treatments (112).

### Alternative Transcripts and Splicing

The human and mouse transcriptomes have multiple ways to splice a gene together through the differential inclusion of exons in the mature RNA. In the 2025 Gencode release for biotypes of RNA, lncRNA, and protein coding have the most transcripts per gene in mice and humans (Fig. 1*B*). This is followed by retained-intron and nonsense-mediated decay RNA transcripts. Breakdown of each protein-coding gene shows low correlation between the number of transcripts annotated in the human and mouse, suggesting that splicing biology knowledge or biological roles differ between the two species (Fig. 1*C*). Short-read sequencing technologies powered the detection of different transcripts at the exon-exon junction detections, limited by the distance of multiple junction sites to know the actual final RNA sequences (39, 113, 114). Genes such as *KRAS* (115, 116), *RAC1* (117), *FGFR2* (118), and *Titin* (119) were well documented for how changes to the protein from splicing altered physiological processes.

The advent of long-read sequencing paired with high depth of sequence coverage has enabled more extensive full transcript maps of gene splicing (120, 121). Integration of both tools with large-scale expression databases enables more systematic maps of splicing, such as the small GTPase family of proteins, including KRAS and RAC1, that have been shown to have a broad protein-altering landscape with phenotypic functions (122). There is an urgent need for basic protein characterizations of splice variants (123). Knowledge of the alternative splicing, including the presence of cryptic splice sites less often used (124, 125), has given rise to the use of antisense oligonucleotides to control splicing around genetic variants in numerous genes such as *SCN1A* (126, 127). The FDA has authorized oligonucleotides that can alter the splicing of genes such as *SMN2* to counter the *SMN1* genetic variants in spinal muscular atrophy (SMA) (128).

The knowledge of the complexity of the spliceosome has significantly increased in the past few years, yielding insights into the many genetic and environmental factors that can regulate the alternative splicing of transcripts (129–133). In many cases, the alternative splicing results in unproductive transcripts regulated by mechanisms such as NMD (134). For example, mutant p53 can result in alternative splicing of multiple pancreatic ductal adenocarcinoma oncogenes (135). Broad splicing changes have been identified within mouse models of ischemic reperfusion injury (91).

There is a complex interaction of splicing factors with polyadenylation machinery as a polyA signal is encountered by the RNA polymerase II (136, 137). A delicate balance of splicing factors’ with polyadenylation factors’ binding (U1 snRNP, SRSF3, SRSF7, CPEB4, CELF2, FUS, HNRNPC, PABPN1), along with the elongation rates and secondary structure of transcripts, can influence the detection of the polyA signal sites within RNA polymerase II transcription to influence the dynamics of exon inclusion and where polyA tail addition ends transcription (137–140). For many transcripts, variable sites in the 3′UTR can be used for the generation of polyA addition and transcript elongation termination, resulting in the same protein but with changes of the 3′UTR regulation of transcripts, including well-known roles in RNA half-life (141). Physiological examples of this include cold exposure dynamics in mitochondrial proton uncoupling in brown fat (142), cell cycle progression regulated by polo (143), and axon-specific targeting of mRNAs in neurons (144), or in many steps of the circadian clocks (145). In other cases, the inclusion of an early exon with a polyA signal site detected by the polyA machinery can result in alternative transcripts, protein isoforms, or even nonprotein coding outcomes such as rapidly degraded transcripts of non-stop decay that can often be impacted in physiological conditions such as cancer (138, 146).

Splicing is not the only biological process that can contribute to differential exon inclusions. Mechanisms for altering either end of a transcript, such as alternative transcriptional start sites and 3′UTR readthrough, can change the processed RNA. The alternative ends of transcripts drive a high diversity of RNA across human tissues (147). The deep sequencing of the 5′UTR as part of GENCODE, ENCODE, and FANTOM consortiums resulted in the identification of alternative first exons of genes, suggesting that within many genes of the genome, there were more than one loci that transcription can start (38, 148–150).

One of the most well-characterized alternative transcriptional start sites in physiology is the *SHROOM3* gene (151). A significant GWAS loci for chronic kidney disease falls near *SHROOM3*, which was annotated for years as intronic within the *SHROOM3* gene (152). However, *SHROOM3* utilizes a secondary transcriptional start site within the kidney podocytes that loops with the upstream GWAS loci (151). CRISPR-based mutation of the CKD risk variant within cell lines directly altered gene expression levels of the secondary start site, and the shorter protein isoform was shown to recover kidney function in morpholino-based knockdown with human mRNA recovery in zebrafish (151). This example is a valuable lesson to carefully annotate noncoding variants relative to RNA transcripts to account for the complete insights into transcript diversity.

Variability in the 3′ end of RNA molecules contains occasional read-through, where the normal 3′UTR gets spliced to an exon that retains an open reading frame. Leptin receptor (*LEPR*) shows the evolutionary selection of usage for RNA read-through, where in vertebrates, the *LEPR* inserted 3′ the endospanin gene (*LEPROT*), utilizing the *LEPROT* broadly expressed promoter to drive ubiquitous expression profile of *LEPR*. One of the most biologically active RNA read-through examples is that of the insulin (*INS*)-*IGF2* fusion transcript, where the read-through of *INS* allows the splicing to join with *IGF2* (153). The *IGF2* 5′UTR uses an alternative open reading frame, resulting in a protein broadly expressed in the pancreas and liver, with human variants further disrupting the stop codon location (154). Variants through GWAS connect this *INS*-*IGF2* transcript to antibody production against Insulin that is highly associated with type 1 diabetes (155).

### Care in Processing Protein-Coding RNA Transcripts

Many studies performing bioinformatics of RNA sequencing sum transcript level mapping into gene annotations, adding all transcripts and protein isoforms into a single gene event. This summation makes gene ontology easier but ignores the biological complexity of transcripts. For genes coding for multiple protein sequences based on alternative exon splicing, a gene expression summation calculation loses the capacity for understanding the transcript-level biology. Further complicating this summation is that of the current protein-coding genes of humans and mice, many contain additional non-protein-coding transcripts, such as retained introns and NMD (Fig. 1*D*). As those transcripts map overlapping segments with the protein-coding isoforms, they can significantly impact the interpretation of protein levels when summing transcripts. We recommend analyzing RNA sequencing data at the transcript level, with biotype-level summation if necessary, per gene to account for these confounding factors.

## SAMPLE ISOLATION AND SEQUENCING PLATFORM COMPARISONS

**Figure 2.**
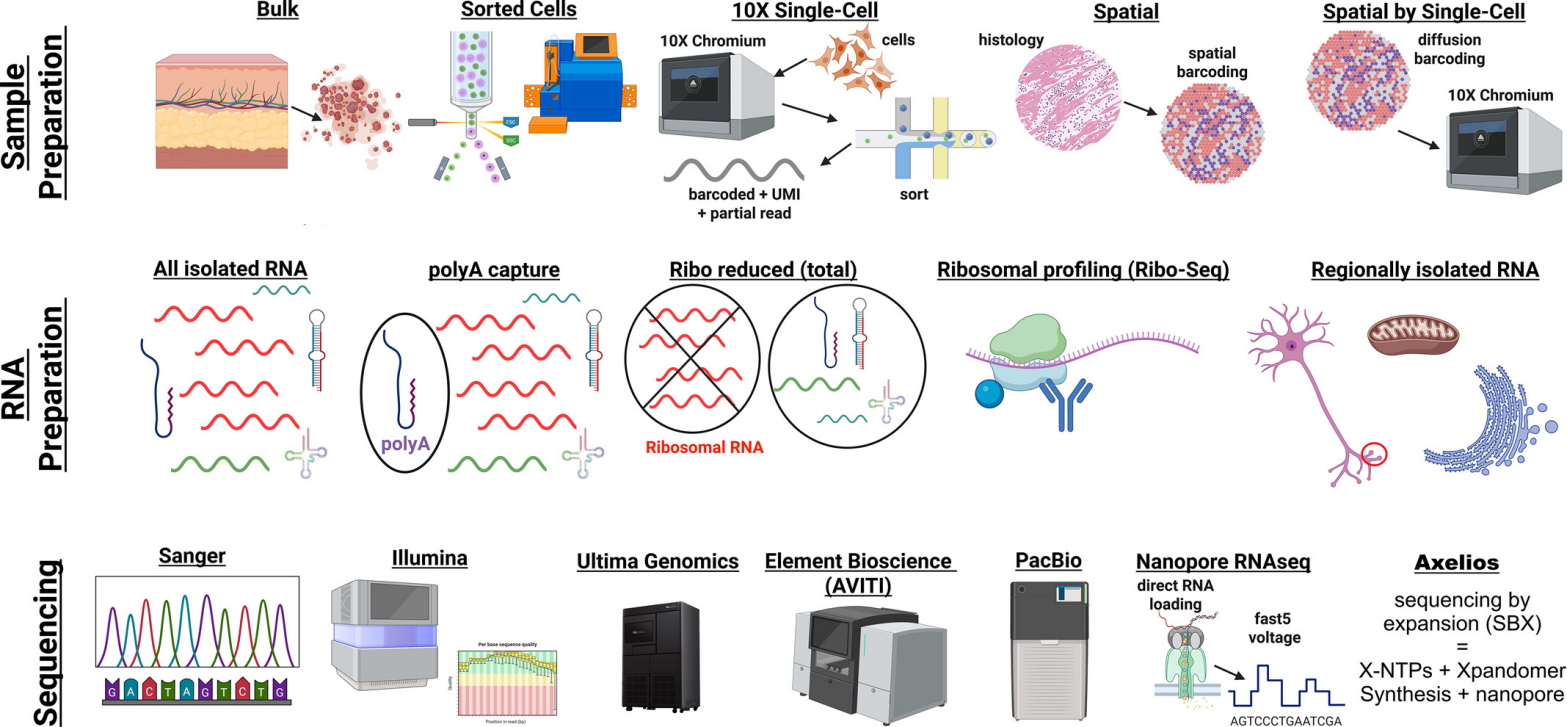
Current state of RNA sequencing techniques. Figure created with a licensed version of BioRender.com.

Over the last decade, the rapid growth of sequencing and the availability of reference genomes have led to the expansion of sample isolation and sequencing methods for analyzing RNA molecules. This has resulted in a set of tools for physiologists, each with benefits relative to the others for sample isolation, RNA preparation, and sequencing of RNA (Fig. 2). Below is a description of each approach/platform, its benefits, and the current challenges, based on the state of knowledge as of 2025.

### Sample Isolation

#### Bulk extractions.

Biomarker discoveries and knowledge advancements in RNA-seq have been driven by the bulk extraction of RNA from whole tissues. Any whole organ or biofluid (blood, saliva, sputum, buccal swab, urine) collected can be lysed and RNA isolated. As RNA isolation became simplified through commercial kits, the speed and efficiency of RNA isolation decreased the likelihood of changes in transcriptomic signatures that can occur in more laborious and time-consuming isolation methods. Moreover, many of the components in between cells that contain RNA (bacteria, exosomes, or cell-free RNA) are also captured in bulk extractions, as presented later in usage of rna sequencing. Bulk extractions blend the many cells that make up tissues and biofluids, creating uncertainty about where signals are emerging and decreasing the ability to map low-cell-number biological contributions. As our knowledge of diverser cell types has expanded, it has created computational approaches for cell type deconvolution of bulk analyses (156, 157). Several computational techniques have been described for dissecting cellular content directly from expression profiles of mixture samples and deconvolving RNA admixture into their component cell types without physical dissociation, antibodies, or living material (158). However, these methods still suffer from signal-to-noise dynamics, uncertainty of the ground truth of cell-to-gene levels, and are often difficult to interpret in light of the limited response biology known at the cell level (159).

#### Sorted cell.

Although the bulk analysis has given broad insights into physiology, the development of single-cell resolution of RNA has given more power to the diverse cell types within specimens. Beginning from the mechanical separation of cells into wells using techniques such as surface capture or flow sorting into high-throughput plates, followed by cDNA preparation, the techniques of single-cell RNA sequencing have now massively parallelized the isolation and preparation of thousands of cells within each sample (55, 160). Early separation of cells from tissues would require extensive time to dissociate material, isolate different cell types, and then perform RNA isolation and sequencing. This yielded early and powerful insights into contributions of different cell types, but was limited to already existing knowledge of what cell groups were, as there was a need to isolate each cell type with known markers. Also, the bulk assessment of any cell group removed much of the heterogenous dynamics, such as cell cycle and feedback loops, being hampered by many of the same challenges found within bulk sample isolations.

Platforms such as the 10× Genomics chromium controller utilize microfluidics to separate cells or nuclei with labeled beads to apply barcodes and unique molecular identifiers (UMIs) to each cell’s RNA, followed by sequencing and deconvolution. As the technique has taken off in applications, the bioinformatic pipelines have simplified the complex analysis, elevating the resources of limited sequencing depth and cell identifications (54, 161). The density of cells sampled enables single experiment landscapes of cell dynamics that facilitate the complex discovery of heterogeneous and homeostasis biology; yet, this complexity has been challenging to understand with the volume of data (162–164).

Single-cell RNA sequencing has revolutionized the identification of cellular landscapes, from cell cycle dynamics to subclusters of functional cells (53). Cardiovascular systems (165), blood cells (166), developmental biology (167), angiogenesis (168), respiratory (169), cancer (170), to nearly every aspect of physiology (171) single-cell RNA sequencing is expanding our knowledge. Compared with other platforms, single-cell RNA sequencing allows exploration of cell heterogeneity at a single-cell resolution, fate mapping, and lineage tracing without genetic labeling, identifying new or rare cell populations based on transcriptomic signatures, and tracking transcriptomic changes in individual cells during disease progression, treatment, and recovery (172). The tools of single-cell are especially powerful for the study of diverse model organisms and environmental factors (173). One of the most expansive usages of single-cell technologies is within the immune system, opening the door for exploring the diverse cell types of innate and acquired activation while observing the combination of chains within the immune repertoire (174–176).

In oncology, single-cell sequencing has led to a better understanding of complex tumor cell behavior, evolution of the cancer, and the identification of novel biomarkers that can be used to develop precision therapies. One significant benefit of single-cell RNA sequencing is improved analysis of multifaceted tumor microenvironments and inferences of cell-cell communication that can assist in creating immunotherapy strategies, particularly overcoming immune resistance (177). More recently, single-cell RNA sequencing has been integrated with other sequencing methods such as ATAC-sequencing for gene regulation (178, 179) to electrophysiology (180) and patch clamping (181) techniques to decipher cellular physiology of gene expression.

Even though single-cell RNA sequencing holds incredible promise, it has several limitations. Most notable is the cost. Although the cost of sequencing each cell type has been reduced to acceptable levels, the platform is still significantly more expensive than bulk sequencing due to the number of cells needing analysis (172). Cells must be handled longer than bulk approaches, increasing the chances that cellular states are altered. Dissociating and sorting cells takes time, which can modify the expression profiles of cells, yielding some nonbiological outcomes. In addition, many cells are impacted by flow-sorting or contain multiple nuclei, such as neutrophils, skeletal muscle, and cardiomyocytes, making it difficult to interpret the full spectrum of cellular/nuclear heterogeneity. Newer strategies are emerging that enable non-fluidic barcoding, lowering the cost per cell.

One of the major challenges with single-cell sequencing is the depth of sequencing needed to quantify expression differences between individual cells. Most laboratories utilize depths of approximately 50,000 reads per cell, elevating to millions of reads when profiling thousands of cells in an experiment (50,000 reads/cell × 10,000 cells = 500 million reads), which is sufficient to identify most cell types with clustering analysis (182). If the focus of a project is rare cell types or differential expression analysis, more robust expression may be warranted (∼100,000 reads/cell). If the focus is on transcript splicing dynamics, even deeper sequencing is suggested (∼500,000 to 1 million reads per cell), quickly elevating the cost to staggering levels relative to bulk RNA workflows. When addressing genes expressed <20 transcripts per million (TPM) in bulk analysis (20 TPM = 1 transcript for every 50,000 reads sequenced), it is often suggested that deeper sequencing is required, but rarely used throughout the field (183). When low sequencing depth is used for each cell, a value of zero has uncertain results and can lead to biased interpretations when reads do show up. Although imputation methods can resolve the technical versus biological zero low-abundance issues, they can also present biases in discovery with overinterpretation or bias of the zero values (184).

Another limitation is that single-cell RNA sequencing only provides static RNA expression profiles, providing limited temporal information and requiring repeat assays to get profiles at different points, such as disease progression and pre- and posttreatment. Pseudotime analysis, a computational tool that ranks potential dynamic processes in cells based on the heterogeneity of transcriptional expression levels, is commonly used in single-cell RNA sequence analysis to create a trajectory mimicking how cells change over time. Continued advancements in algorithms, computational tools, and multiomics can reduce the limitations (185). Finally, the nature of 5′ or 3′ short-read sequencing, often used in single-cell does not resolve full transcript dynamics, making many of the above discussions of biotype and transcript discoveries challenging (186). The pairing of single-cell sequencing with long-read chemistries promises to remove some transcript limitations but further elevates the cost per cell for data, given the required number of reads per cell to accurately measure transcript differences (187, 188). Overall, single-cell RNA sequencing advances our knowledge of cellular physiology and will continue growing to overcome many limitations.

#### Spatial sample processing.

Although single-cell RNA sequencing advances cellular discovery, spatial RNA sequencing promises to refine our tissue physiology and disease knowledge (189, 190). The complex anatomy of cells within any biological tissue has long been appreciated through histology, and dating back to 1987, groups have advanced the mapping of genes into spatial discoveries (191).

The rapidly emerging technologies to transition histology into RNA sequencing have powered spatial transcriptomics growth. Most notably, utilizing spatial sequencing with specialized computational tools such as reference single-cell RNA sequence atlases, cell2location, cytoSPACE, ST deconvolve, and Boyers space enhances cellular resolution, transcript coverage, and alignment of independent samples (189). For example, surfaces can be labeled with barcodes, and the histology sections laid down on that surface to facilitate spatial barcoding that can be deconvoluted post-sequencing. The density of these barcodes has elevated to reach a sub-single-cell level map of expression. These advancing technologies have yielded unparalleled insights into physiology’s spatial heterogeneity and homeostasis (192). They have also validated the identity of new cell types discovered by single-cell sequencing, expanding cell-cell or neighborhood relationships within functional tissue units, and generated cellular and molecular atlases of healthy organs through the work of groups such as Human Biomolecular Atlas Program and Human Cell Atlas (193). The application to heart (194), kidneys (193, 195), skeletal muscle (196), endocrine organs (197), skin (198), brain (199, 200), and lung (201), has grown basic knowledge of healthy tissue with more recent applications to disease pathologies.

Similar to all techniques, spatial transcriptomics has multiple limitations, most notably the cost per sample processed. The limited sequencing depth of spatial platforms can also make the depth of cell heterogeneity difficult. Pairing single-cell RNA sequencing enables a higher sequencing depth for cells matched to histological locations of spatial transcriptomics. This shows that technique integrations hold the greatest depth of knowledge advancement but with significant cost limitations (189). One of the significant limitations of spatial transcriptomics is the need for cells to fit into histological slides, where many tissues create challenges. Similarly, artificial intelligence digital pathology tools such as FUSION cannot perform the same interpretation as manual work by pathologists in providing histological correlation to spatial transcriptomics and cannot provide single-cell resolution. As a result, the time and cost prohibit the process of having pathologists manually annotate spatial transcriptomics data for accurate results (193). The three-dimensional nature of tissues also elevates the cost of spatial transcriptomics with the need for many slice analyses to recreate the maps of whole tissues, limiting the work to well-funded consortium initiatives.

Emerging tools are combining spatial mapping with single-cell technologies. Tools such as Curio Trekker use spatial barcodes that are released from the surface to diffuse into and label the nucleus that is closest (202). When the nuclei are dissociated, it enables single-cell sequencing that can be taken back to the spatial barcode locations.

Most spatial transcriptomics has been performed on mRNA with limited accounting for the noncoding biotypes of protein-coding genes (58). Similar to single-cell sequencing, the technological pairing of spatial with long-read sequencing holds the promise to move to transcript-level discoveries (194). Still, it also significantly elevates the cost of an already expensive technique. Spatial transcriptomics, although still limited in its infancy, most likely holds the greatest potential of all RNA sequencing platforms for the field of anatomy and physiology and will likely bring critical advancements to the characterization of many underexplored genes of the human genome. As new spatial platforms such as the Aviti24 Teton cytoprofiling technology emerge, we will also see the fusion of multiomics, where proteins can be detected using barcode-labeled antibody binding sequencing linked to RNA sequencing within the same spatial samples.

### RNA Preparation

#### Bulk RNA.

Sample preparation is not the only part of the isolation process that alters the discovery potential in RNA sequencing. The type of RNA molecules isolated also has significant impact on how data are interpreted. The isolation of all the RNA within the cell, followed by sequencing, results in the majority of data to be that of rRNA. The resulting data from bulk RNA isolation produces only a fraction of usable insights and requires deeper sequencing to find valuable signals. Thus, there is a need to isolate RNA types or to remove the rRNA to identify biologically meaningful information.

#### PolyAdenylation capture.

Given that eukaryotic protein-coding RNA is polyadenylated, it is possible to capture these specific RNA molecules using the polyA signal to remove the rRNA. This can be accomplished through the use of oligo dT sequences to initiate cDNA amplification, through the use of labeled beads enabling mRNA capture, or in some cases the engagement into protein pores in platforms such as nanopore (203) (where bulk RNA can be used as starting material). It should be noted that prokaryotic species RNA lacks polyA tails and thus cannot be captured with this method. The capture of polyA mRNA also removes most of the other noncoding RNA biotypes in addition to rRNA. Although our knowledge of these other RNA biotypes has been slower to discovery than mRNA, as already addressed, their increasingly recognized importance has shifted the RNA sequencing field to pivot more toward other isolation methods, even as polyA isolations are a far cheaper method of removing rRNA.

#### Ribosomal reduction.

To remove the rRNA, which accounts for 80%–90% of cellular RNA, techniques have emerged to deplete rRNA through selective hybridization (204). The remaining mRNA and the diverse non-coding RNA can then be sequenced on any sequencing chemistry. These ribosomal (ribo) reduction strategies are of high use in prokaryotic species that lack polyA mRNA signalling. Although ribo reduced depletion methods produce robust insights for biotypes of RNA, the isolation cost significantly more than polyA isolations. Newer generation, often species-specific, rRNA enzymatic degradation methods are beginning to reduce the costs of ribo reduction (205, 206).

#### Other profiling techniques.

Several other RNA isolation methods outside of poly(A) capture and ribo reduction have also been developed. Isolation of RNA from organelle-specific locations, such as mitochondria, endoplasmic reticulum, or the nucleus, has emerged (207, 208). Ribo-Seq offers a capture of the active engagement of RNA with the ribosome for translation (50). The capture of ribosome-engaged mRNA also enables kinetic insights into the dynamics of translation (209, 210), with the techniques now moving into single-cell resolution (211). Neurons have the ability to transport mRNA far from the nucleus to individual synapse regions (212). Neurites have one of the most complex localization systems, where protein localization is primarily driven by the mRNA localization, insights made possible through unique neurite RNA isolation methods (213). Interactions of the RNA 3′UTR elements with proteins such as UNK drive the RNA to the neurites (214). Together, these isolation techniques expand RNA-seq beyond abundance toward understanding localization, translation kinetics, and regulation within complex cellular microenvironments.

### Sequencing Platforms

#### Sanger.

The sequencing of DNA has long been done through fragments of ∼1,000 bases, starting with Sanger and automated Sanger platforms and advancing into next-generation platforms. As RNA sequencing increased, many genes were first discovered through the isolation, cloning, and Sanger sequencing of cDNA. Although Sanger sequencing can produce fragments of larger than 1,000 bases with high fidelity, the extreme cost per base relative to other platforms often pushes these platforms more into validation methods or low complexity assays with a few readouts (215).

#### Short-read sequencing.

The short-read platforms were primarily driven by Illumina-based sequencing, with newer companies such as Element Biosciences' AVITI platform and Ultima Genomics UG 100 elevating a new competitive sequencing market. The cost of these machines can represent a significant investment for laboratories, where many institutes and commercial vendors offer sequencing through cores and services to bring the technology to more laboratories. It should be noted that the cost per base of sequencing significantly decreases with the larger volume machines, where pooling strategies are implemented in large volume centers to reduce per-experiment costs.

The foundation of all short-read platforms is the fragmentation of nucleotides, cDNA in the case of RNA sequencing, followed by polymerase addition of labeled nucleotides and some form of imaging or size separation. The sequencers of short-read platforms can take upwards of a day to return any data during sequencing. Short-read sequencing can be performed as either single-end or paired-end. In single-end, one side of the fragmented cDNA is sequenced, whereas in paired-end, both are sequenced. Although single-end sequencing is cheaper and takes half the time to complete, paired-end sequencing is preferred by most laboratories, given the enhanced nature of the reads to confirm splice sites.

#### Long-read sequencing.

The use of long-read sequencing platforms has revolutionized the detection of gene-level transcripts. The two commonly used long-read sequencing platforms are PacBio and nanopore. These long-read techniques have an increased cost per sample relative to short-read platforms, with further increase in cost when paired with single-cell or spatial RNA sequencing workflows. Newer improvements, such as using unique molecular identifiers (UMIs), have increased the accuracy of these long-read platforms by generating consensus around the multiple reads of each UMI, but these approaches increase cost (216). Long-read sequencing has been especially valuable for under-characterized or novel model organism genomics and transcriptomics, where it can generate reference maps with less bioinformatic needs (217, 218). This is particularly important in the rapidly evolving field of metagenomics (219, 220).

PacBio, which sequences cDNA using real-time polymerase tracking known as single-molecule real-time sequencing, has a more considerable capital cost for the sequencer but provides higher quality/accuracy sequencing outputs. The circularization of cDNA constructs enables read corrections through repeated sequencing of each fragment, which can be used for additional single-cell preparations (221). Nanopore, which can sequence cDNA or direct RNA through protein pores to track subtle voltage changes, offers the lowest capital sequencing costs (<$1,000) but has lower sequencing accuracy. Both of these long-read platforms offer full-length transcript sequencing that facilitates the analysis of all transcript splice sites per read, enhancing the identification of splicing complements greater than the few-hundred-base limit of short-read technologies. Similar to short-read technologies, both platforms require additional methods to give cellular or spatial insights. The platforms are also more challenging to handle batching, with the number of reads generated per run far below the short-read high-density sequencing platforms, decreasing the number of samples barcoded into a single sequencing run.

For the nanopore platform, with the laboratory addition of polyA tags onto the RNA and ribo-reduction, it is possible to sequence noncoding RNA. In addition, nanopore is currently the fastest platform for data generation, as the voltage analysis of each read can be rapidly converted into base calls, generating real-time sequencing insights that accumulate over time. In addition, nanopore offers the ability to directly run RNA molecules through the protein pores, thereby reducing the time to data generation without requiring cDNA conversion (222). The direct RNA bases can also be processed for the role of RNA base modifications (223, 224), similar to DNA methylation, but where RNA is known to have dozens more modification types than DNA (225–227). Nanopore’s rapid and direct data generation makes the platform ideal for future scaling into clinical specimen analysis, generating much excitement for applications in physiological genomics. For example, nanopore offers return of that data that could be used when the need for data is exigent, such as in critically ill and presurgical patients.

Although much of the sequencing market has been dominated by Illumina short-read, these long-read technologies continue to mature. Newer possibilities, such as sequencing by expansion (SBX) chemistry of Roche (228), are completely redesigning the strategies of sequencing. SBX utilizes novel expandable nucleotides (X-NTP) bases that can be added to synthesis using Xpandomer methods, creating bulkier bases that are rapidly and accurately sequenced at ultra-low cost through nanopore structures. As we enter the new era of a competitive sequencing market, there is considerable hope that competition will drive innovation and cost reductions for RNA sequencing, thereby bringing many of these tools to a wider range of laboratories.

## RNA SEQUENCING RESOURCES

### Publicly Available RNA Sequencing Data

**Figure 3.**
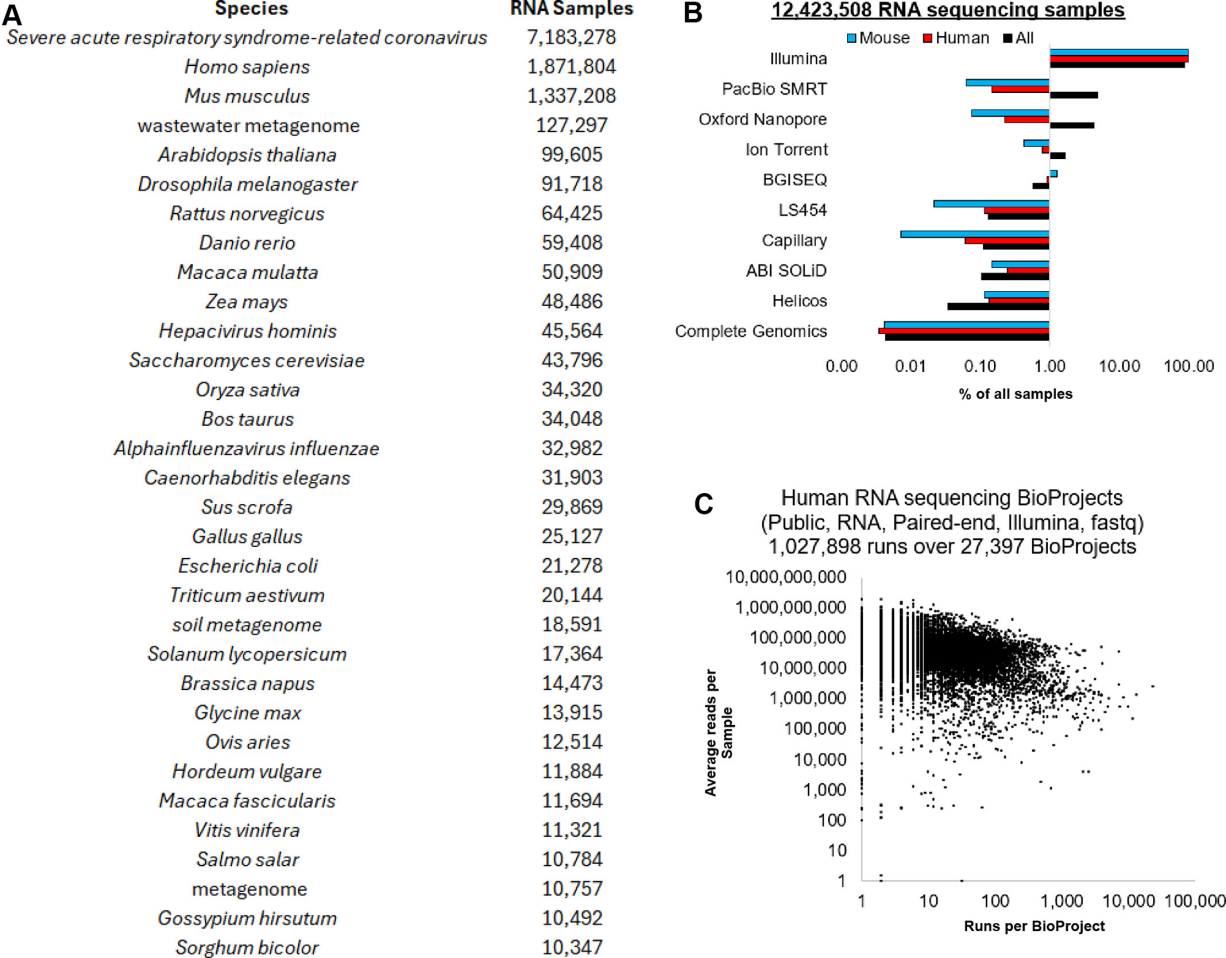
Data for RNA sequencing available in the NCBI SRA. *A*: number of RNA sequencing runs per species. *B*: the chemistry types of RNA sequencing for NCBI SRA runs. *C*: the number of runs within a BioProject (*x*-axis) and the average depth of reads (*y*-axis) per sample of RNA sequencing datasets. All data was pulled on November 11, 2024.

Millions of samples have been processed as the above RNA sequencing platforms have been utilized for the past two decades. Many of these datasets are available within public databases, enabling scientists to reprocess data as tools and resources grow. For example, as discussed in the human genome transition to transcript discovery, as the Gencode knowledge has increased over time, reprocessing older data can provide insights into transcripts not annotated within older Gencode releases. The National Center for Biotechnology Information Sequence Read Archive (NCBI SRA) contains the largest deposit of raw sequencing data. As of late 2024, the species with the most datasets available is SARS-CoV-2 (Fig. 3*A*), an RNA virus sequenced extensively during the 2020 pandemic (229). Human RNA sequencing datasets are approaching 2 million available runs, with mice also above a million datasets. This is followed by a rapid decrease in data for other model organisms, such as plants, flies, rats, and fish (Fig. 3*A*).

Most sequencing has been on the Illumina platform for the 12 million runs of RNA sequencing available, with a rapid decrease to PacBio and nanopore-based long-read datasets (Fig. 3*B*). It should be noted that human and mouse long-read datasets are far below other organisms, such as SARS-CoV-2, for long-read sequences. For humans, Illumina datasets available for public download as the FASTQ file and paired-end, there are over one million sequencing runs from 27,397 projects (known as BioProjects within NCBI) at the end of 2024 (Fig. 3*C*). These projects contain a broad range of sample and sequencing depths. The sequencing depth of any experiment represents the number of reads generated per sample, where more reads increase the confidence of data mapped to a reference as coverage of unique splice sites increases, especially for RNA molecules with lower expression levels. If analyzing gene transcripts, splicing, the immune repertoire, or environmental factors (bacteria or viruses), samples should be sequenced to a higher depth. When interested in quantifying human mRNA, many groups will euthanize the sequencing depth to elevate samples and stretch the cost of sequencing.

Many large consortia deposit vast resources of raw RNA sequencing datasets such as ENCODE (BioProject PRJNA30709), the noncoding RNA atlas (PRJNA576920), and the Precision Medicine Research consortium (PRJEB58625). In some cases, the RNA sequencing data is protected under the database of Genotypes and Phenotypes (dbGaP), where Institutional Review Board (IRB) and safety plans are required before data can be obtained to protect human specimens from reidentification using the raw reads.

The depositing of data for single-cell datasets is unstructured in the current NCBI SRA, with some groups deconvoluting data before submission, resulting in a large number of runs with low depth of coverage, such as BioProject PRJNA591860 and PRJNA638640. In other cases, runs with deep sequencing can often be single-cell datasets deposited without a breakdown of each cell’s expression, such as BioProject PRJNA551745 and PRJNA788872. Groups working with any NCBI SRA RNA sequencing data are advised to read as much information as possible on the breakdown of runs and sequencing depth before working with the data. This is also why including detailed metadata when depositing samples into the NCBI SRA is so important.

In many data analysis projects with already generated RNA sequencing datasets, laboratories must account for batch effects and the experimental clustering of data, including experimental design, RNA extractions, and sequencing methods. Batch effects can be normalized with various computational methods, but none of these methods are perfect, and care should always be taken when comparing quantitative data across BioProjects (230–232). In data analysis from multiple BioProjects, selecting desired data quality, sequencing depth to reflect the type of molecules of interest, and a larger volume of samples to enhance discovery is normal.

A significant limitation of reprocessing NCBI SRA data is the need for large data downloads and extensive storage. One of the methods for rapidly identifying desired reads within an RNA sequencing data set is using the SRA BLAST tools (233), where the SRA database can be selected from BLAST search settings. This tool can have utility, for example, in searching unique splice sites from a few select SRA runs placed in the SRA accession. If someone wishes to process full alignments of SRA data, the SRA toolkit simplifies data downloads and parsing of paired-end reads.

To mitigate the issues of massive data downloads for RNA sequencing analysis, the NCBI has built an SRA in the cloud strategy that enables researchers to utilize Amazon or Google cloud approaches to move buckets of data to remote computing. These cloud strategies are suggested for optimal outcomes for projects where thousands of RNA sequencing datasets will be processed. As the utilization of RNA sequencing continues to scale throughout physiology, it is essential to collaborate in data processing to better optimize hypotheses and experimental designs in the context of the extensive RNA sequencing resources available.

### Model Organisms

The laboratory mouse (*Mus musculus*) is the most studied model organism with RNA sequencing. By the end of 2024, the laboratory mouse had 1,349,165 runs within the NCBI SRA, 769,291 as paired-end, and 579,874 as single-end. Almost all these datasets are from Illumina short-read platforms, with around 2,000 samples for long-read technologies.

The mouse genome database contains a large amount of curated resources on RNA sequencing. These include curated and normalized RNA sequencing datasets, developmental biology insights, and differential expression profiles in experiments (234). One of the primary reasons the mouse has been so well explored for RNA sequencing is the many genetic models used for physiological characterizations, including consomic/congenic mapping, recombinant inbred, heterogenous stock, CRISPR editing, and conditional tissue-specific knockout methods. The GeneNetwork tools have curated many of the RNA sequencing resources of commonly used genetic models of laboratory mice, creating an easy-to-search tool for any gene symbol (235).

The zebrafish (*Danio rerio*), fly (*Drosophila melanogaster*), rat (*Rattus norvegicus*), and other organisms all have RNA sequencing datasets available and are curated in their respective genome databases (Table 1). Although these species have thousands of datasets, it should be noted that the transcriptome references have still lagged behind that of the human and mouse annotations based on the limited sampling. These transcriptomes often lack extensive noncoding RNA transcripts, and the protein-coding isoforms are often underdeveloped for diverse tissues and splicing complexity. In a recent study from our team, we demonstrated a 30%–40% difference in alignment for samples of the right or left ventricle relative to the leaflets of the valves within the hearts of sheep (242), highlighting the major limitations in diverse species RNA assessments without establishing de novo transcriptome annotations.

**Table 1.** Model organism databases with RNA sequencing data and resources

Model Organism	Database	Website	Citations
Mouse	MGI: Mouse Genome Informatics	https://informatics.jax.org/	(236)
Rat	RGD: Rat Genome Database	https://rgd.mcw.edu/	(237)
Zebrafish	ZFIN: Zebrafish Information Network	http://zfin.org/	(238)
Worm/Nematode	WormBase	https://wormbase.org/	(239)
Frog	Xenbase	https://www.xenbase.org/	(240)
Pig	Pig Genome Database	https://www.ensembl.org/Sus_scrofa	
Dog	Dog Genome Database	https://www.ensembl.org/Canis_lupus_familiaris	
Sheep	Sheep Genome Database	https://www.ensembl.org/Ovis_aries	
Cat	Cat Genome Database	https://www.ensembl.org/Felis_catus	
Cross-species	Alliance of Genome Resources	https://www.alliancegenome.org/	(241)

Many other model organisms have had their genomes sequenced and paired with RNA sequencing to annotate transcriptomes available within the NCBI genome resources. Translational research studies often leverage large animal models to improve translation into human disease insights, and while databases do exist for many of these large animal models, such as pig (*Sus scrofa*) and sheep (*Ovis aries*), annotation is much more limited, and breeding is much less controlled. Care should be taken when working with any model organism to address whether the reference resources included tissues selected for experiments. Otherwise, it is advised to consider assembling a de novo transcriptome using tools like Trinity (243) or performing long-read sequencing for better transcript maps. There is incredible potential for expanding RNA sequencing to diverse animal model physiology, as our team has shown in using RNA sequencing to uncover unique gene expression in the tricuspid valve due to pulmonary hypertension (244), discover the leptin sequence in birds (245) or to define genes on the rat sex chromosomes (246, 247).

### Variant to Gene Tools

**Figure 4.**
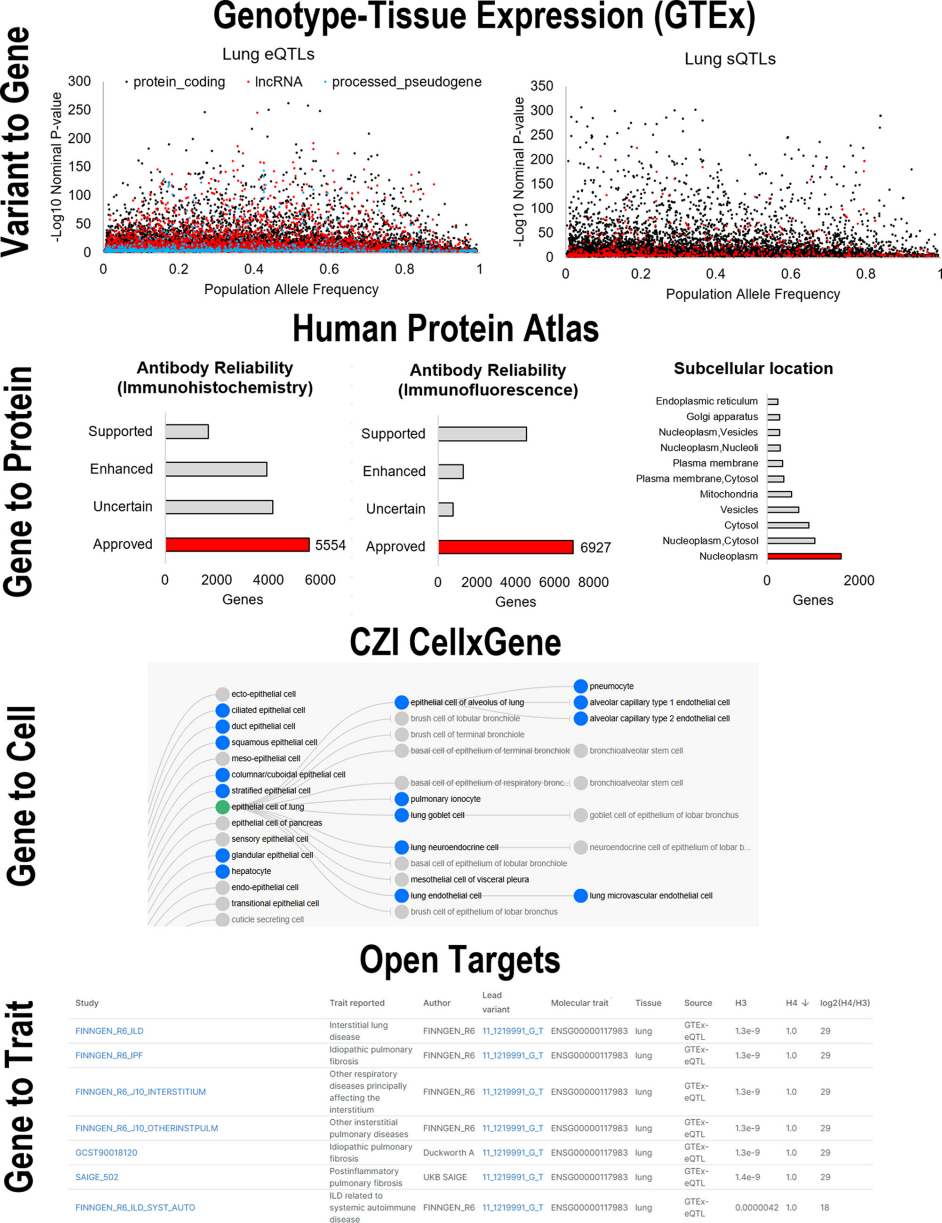
Databases of RNA sequencing to physiological mechanisms.

As millions of human RNA sequencing data points are available, integration with other physiological tools has expanded (Fig. 4). One of the most valuable resources has been linking genomic data to gene expression (variant to gene, Fig. 4). Over a million human genomes have been sequenced, informing population structure and rare variants (248–250). The gnomAD database, as of version 3 in 2024, contains 730,947 exomes and 76,215 whole genome sequences. This population structure and frequency of variants have been linked to large-scale RNA sequencing analysis of 54 non-diseased tissues from approximately 1,000 organ donors to build the genotype-tissue expression (GTEx) consortium (251, 252). Genetic variants have been associated with expression differences within each tissue, known as expression quantitative trait loci (eQTLs) or splicing quantitative trait loci (sQTLs) differences . The vast resources of GTEx can be studied through an online browser for genes or variants, providing integrated statistical analysis of *P* values and fold change for any tissue that can be compared with population allele frequencies (variant to gene axes, Fig. 4). Multiple ongoing consortia are moving eQTL analysis into single-cell RNA sequencing data integrations.

The expansion of ENCODE datasets can further refine eQTL variants into biological mechanisms, particularly for gene regulation (253). Simplified analysis can be performed using genome annotation states such as ChromHMM (254) and simplified scoring tools such as CADD (255). Care must be taken when linking any quantitative trait loci to mechanisms of gene regulation, as the inheritance of multiple variants within linkage disequilibrium (LD) complicates trait analyses, often requiring narrowing multiple potential causal variants for eQTLs (151). Polygenic priority score is a gene prioritization method that leverages GWAS summary statistics and incorporates data from bulk and single-cell expression datasets, curated biological pathways, and predicted protein-protein interactions (256).

### Gene to Protein Tools

Broad human RNA sequencing profiles can also be linked to protein and spatial biology by integrating expression with proteomic techniques such as immunohistochemistry, immunofluorescence, and subcellular localization analyses (Gene to Protein, Fig. 4) (257). The vastest resource for linking these datasets is the Human Protein Atlas (HPA) (258, 259), consisting of thousands of highly curated and validated antibody studies to link genes to proteomic resources. These online tools have grown to include brain analyses (260), functional classes of immune cells (261), secreted proteins (262), single-cell datasets (263), The Cancer Genome Atlas biomarkers (264), and commonly used cell lines for tissue culture (265). These HPA resources are valuable tools for physiologists to link gene expression to thousands of proteins.

### Gene to Cell Tools

As single-cell and spatial platforms have emerged as powerful cellular and tissue resources, they have also resulted in organized consortia to pool resources into valuable databases (Gene to Cell, Fig. 4). The most complex of human tissues, the brain, highlights the incredible growth of these consortia. The Allen Institute’s investments in organizing brain data have given rise to the resources of the Allen Brain Map (266, 267). From complex circuits to connectivity, the brain resources have been merged with RNA sequencing resources to inform mouse and human developmental biology and disease genetic architecture. The larger consortia outside the Allen Brain Atlas have furthered these tools into a systematic map of the human brain connectome that was heavily influenced by RNA sequencing discoveries (268, 269).

The Chan Zuckerberg Initiative (CZI) has heavily invested in community data integrations for single-cell and spatial profiles of nearly every human organ (Gene to Cell, Fig. 4). Their CellxGene tools comprise over 100 million cells from >1,700 datasets for differential expression, spatial maps, cell developmental trajectories, and annotated cell expression (270). The integrated analysis of these datasets has elucidated nearly a thousand functional cell types based on the expression of unique genes that can be integrated into any existing or future single-cell RNA sequencing experiment to facilitate cell annotations.

### Gene to Trait Tools

The most crucial aspect of any physiologist’s research program is the exploration of phenotypes and disease traits, where RNA sequencing can be integrated with the scaled exploration (Gene to Trait, Fig. 4). The gap in genotype to phenotype is the major obstacle in advancing RNA signals as biomarkers in clinical decision-making. There have been attempts, mainly in oncology, to advance phenotype to genes through RNA profiles to identify genes linked to cellular transformation (271).

The first knockout mouse model altered the immune-related gene β2-microglobulin to drive a deficiency in CD8 T cells (272). Since then, thousands of laboratories have knocked out genes in species including mice, rats, and zebrafish, followed by phenotyping. With higher throughput approaches to mouse knockout studies, thousands of genes can be linked to high-density programs such as the International Mouse Phenotyping Consortium (IMPC). With over 100,000 phenotypic statistical tests from nearly 10,000 gene knockouts, the IMPC also provides knockout replacement strategies to explore gene expression during development (273). The rat genome database also provides hundreds of gene knockout and genetic breeding schemes for phenotypic insights (274). Sometimes, the scientific approach neglects what the knocked-out gene does not affect rather than what it does, something that RNA sequencing could further elucidate if paired with approaches such as the IMPC.

**Figure 5.**
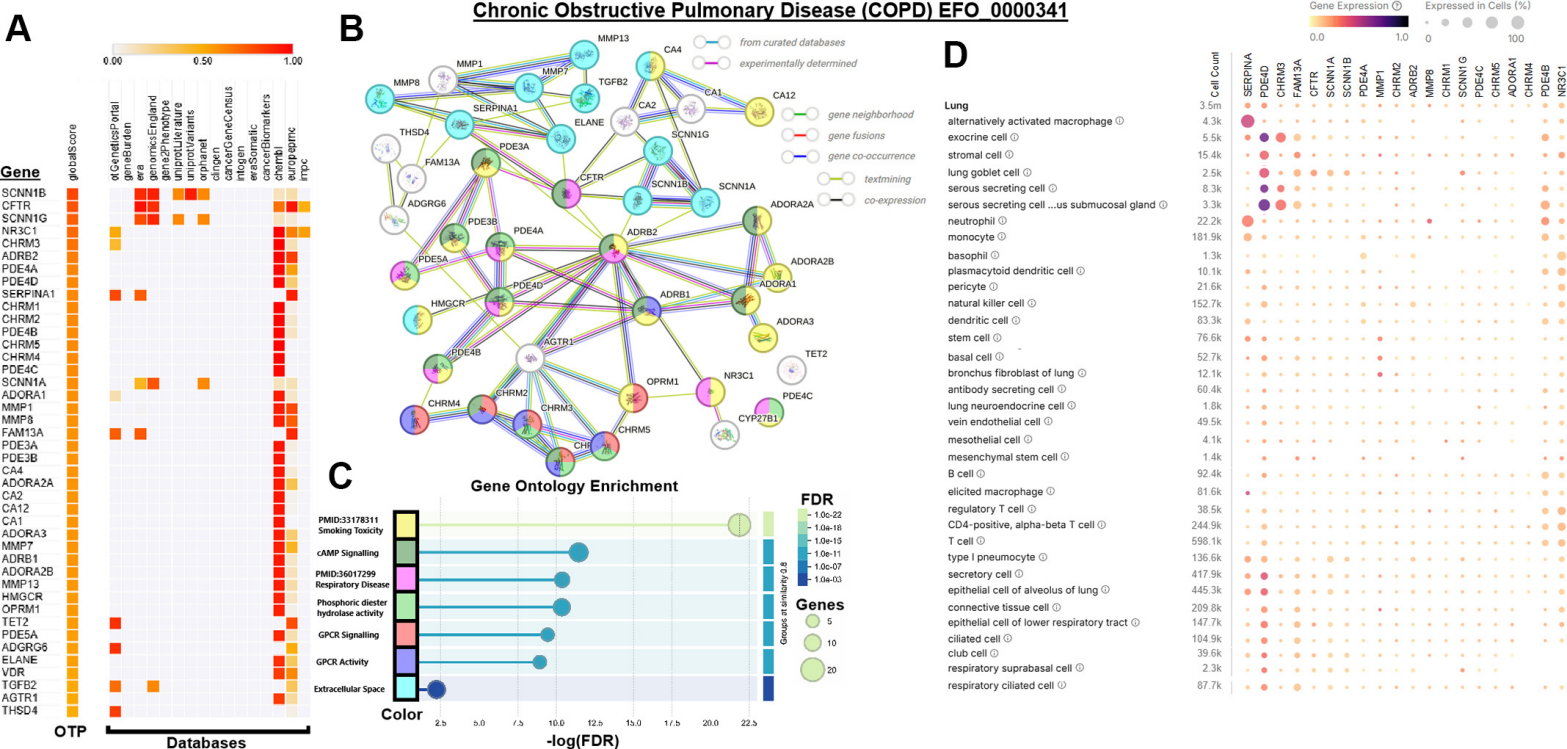
Gene to Trait analysis for COPD. *A*: heatmap of Open Target Platform (OTP) scores >0.5 extracted for chronic obstructive pulmonary disease (EFO_0000341, the unique id for this medical phenotype term). *B*: STRING-based network analysis of *A* genes for human knowledge of protein interactions. *C*: Gene Ontology enrichment terms for *B*. *D*: CZI CellxGene single cell expression for the top 20 genes from *A*.

Human genetic variants have traditionally been linked to physiological traits through either rare disease genetic mapping or Genome/Phenome-wide association studies (GWAS/PheWAS) (9, 275). RNAseq and genetic variant insights can be connected to various cancers through the COSMIC database (276). The GWAS catalog started the large-scale curation of thousands of studies for hundreds of biological traits (277). The UK biobank has furthered this work by colocalizing the variant to gene tools for each GWAS/PheWAS-associated loci (Open Targets Genetics, OTG) (278). These tools allow users to search phenotypic traits, which returns a list of loci that link eQTL/sQTL data from variant linkage disequilibrium (LD) blocks to genes. The data analysis of OTG further integrates multiple gene-centric databases with the GWAS/PheWAS data into a curated list of genes to disease (Open Targets Platform, OTP) (279). For example, it is possible to extract the highly associated genes for chronic obstructive pulmonary disease (COPD) from a dozen data structures into a simple tab-separated file from OTP (Fig. 5*A*). These genes can quickly be processed for pathway analysis using tools such as STRING (280) (Fig. 5*B*) and gene ontology enrichment (Fig. 5*C*) to establish physiological mechanisms for disease. Further dissection using the CZI CellxGene tools can resolve the functional cells within the lung tissue, each gene may work through (Fig. 5*D*). This workflow has available data for thousands of physiological traits and biological questions.

A recent study using an ancestry GWAS/PheWAS approach identified new putative causal variants, genes, and pathways, some targeted by existing drugs (281). As the All of Us data structure grows for nearly a million genomes linked to medical insights, further discoveries linking RNA sequencing to biological traits will be made. GWAS and single-cell RNA sequencing can substantially improve our understanding of disease heterogeneity and the identification of COPD (or other disease) endotypes and subpopulations (282).

### Current Challenges within RNA Data

The massive expansion of RNA sequencing in physiology is not without continuing challenges. As already discussed, there is a need to define the physiology of noncoding RNA, where new techniques and the expansion of CRISPR-based technologies hold promise but require extensive new funding initiatives. More simple challenges within the data can also be seen. For example, existing RNA resources (Fig. 3) highlight the imbalance of RNA sequencing resources for large animal models more closely resembling human physiology. Models such as pigs, sheep, and cattle should be further explored for bulk tissue, single-cell, and spatial analysis through strategically invested research funds.

**Figure 6.**
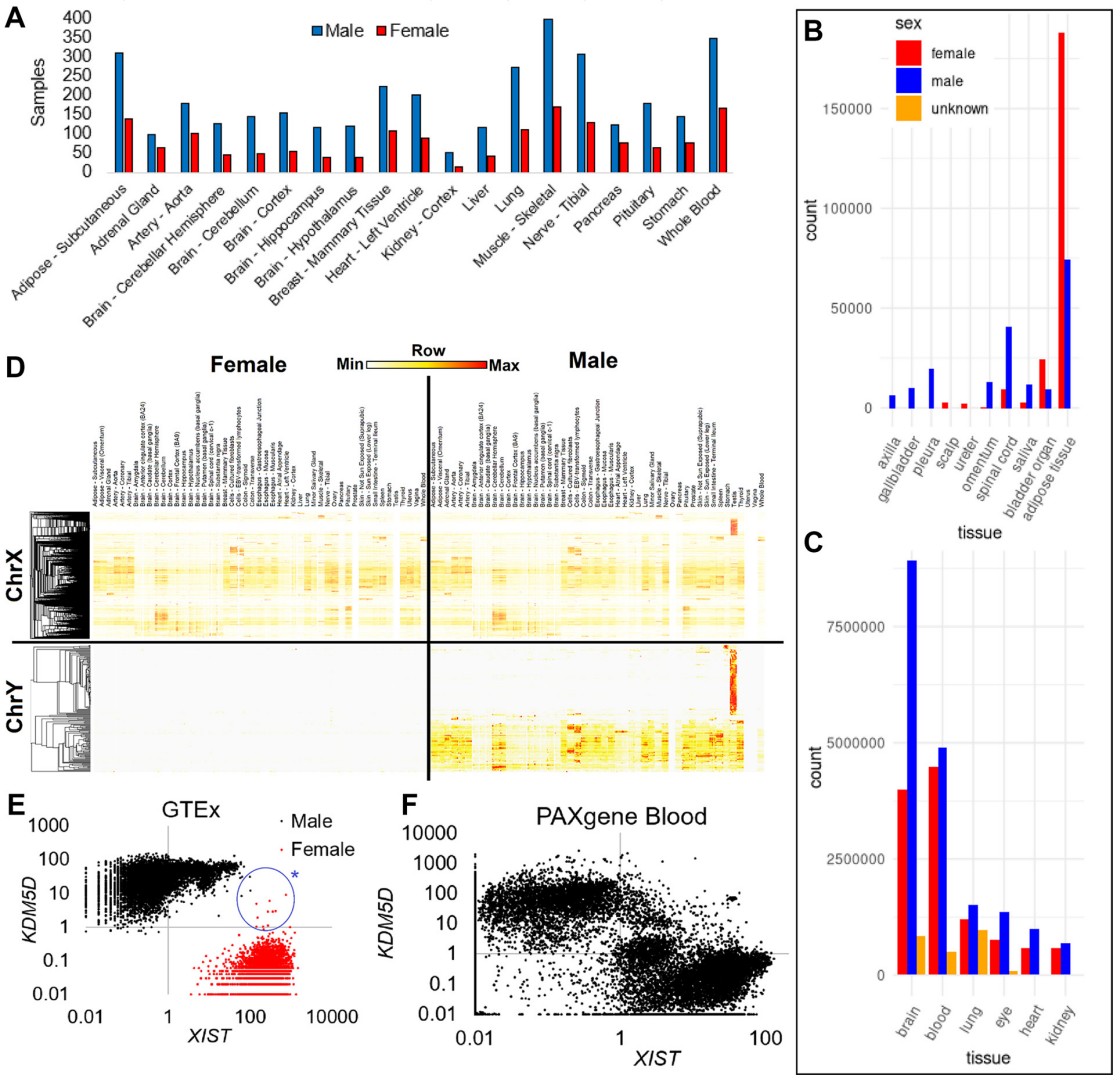
Sex biases of sample collections within large RNA sequencing databases. *A*: the number of samples within GTEx tissue RNA sequencing for male (blue) or female (red). *B*: ten human tissues in the CZI CellxGene Census have the largest imbalance by sex (excluding sex-specific organs). Five samples were from only one sex, and the remainder had a ratio greater than 2:1. *C*: tissues with more than 1 million cells in the CZI CellxGene census show a consistent bias toward male samples, particularly in brain tissue with additional complexity due to the presence of cells with no sex listed as notable in the lung. *D*: heat map of the ChrX or ChrY transcripts from GTEx in males and females. Each tissue is averaged over multiple samples. Each row shows a *z*-score with red being the highest tissue for that transcript. Dendrograms represent one minus Pearson’s correlations. *E* and *F*: *KDM5D* of ChrY (*y*-axis) and *XIST* of ChrX (*x*-axis) expression levels from 17,382 GTEx samples over 54 tissues (*E*) and 16,243 blood PAXgene tube samples (*F*) processed from our previous work (283). CZI, Chan Zuckerberg Initiative; GTEx, Genotype-Tissue Expression.

One of the significant challenges in physiology is the analysis of sex and gender as variables in phenotypes. Datasets such as GTEx (Fig. 6*A*) and the CZI atlas (Fig. 6, *B* and *C*) highlight the imbalance in samples collected between annotated sexes. These imbalances can make it very difficult to dissect molecular contributions to sex and gender, driving often over over-simplified views of what sex and gender are (284). Within sex differences, one of the needed significant expansion areas for RNAseq is the understanding of expression for genes from the sex chromosomes, where the broad tissue atlas of GTEx shows that some genes have ubiquitous expression while others are enriched to specific tissues, such as the reproductive organs (Fig. 6*D*). RNA sequencing has been critical to understanding sex differences by establishing relationships between gene dosage (copy number) and transcript abundance of genes on the sex chromosomes (285, 286). Expression from the Y-chromosome (ChrY) genes has remained one of the most understudied aspects of sex differences (287), where GTEx data suggests many genes show tissue specificity (Fig. 6*D*, *bottom right*) yet lack physiological characterization.

X-chromosome inactivation (XCI) and the escape of XCI by various genes has developed at the single-cell level using variant tracking (288, 289), emerging to show the heterogeneous nature of variant influences on sex-linked phenotypes. More investments into these methods need to be explored. XCI dynamics are often assumed to be an efficient mechanism, as is the presence of ChrY genes. Yet, plotting the ubiquitously expressed ChrY gene *KDM5D* relative to the *XIST* gene for known sexes of samples from GTEx suggests high variability with the presence of ChrY genes and *XIST* elevation (Fig. 6*E*). Further processing of 16,243 blood PAXgene tube samples from our previous work (283) also suggests the same trend observed for *KDM5D* and *XIST* expression (Fig. 6*F*).

Sex, as defined by RNAseq, is not binary, as we show in Fig. 6. Sex chromosome genes can have different states that may reflect concepts of microchimeric sex chromosome genes and cells during pregnancy (290), genetic loss, or gain of sex chromosomes and their genes for intersex disorders (291), or the occasional XCI variability between tissues and individuals (292). RNA sequencing is critical to further refine the nature of XCI imbalance and dosage of sex chromosome genes that can cause female disease for primarily annotated male diseases due to a single ChrX inactivation throughout all cells (293). Gender has been even less explored based on gene expression, as has the understanding of hormone replacement therapies. Recent work has started to show the value of these explorations of RNA sequencing for gender (294). Using transcriptomics, the individual’s sex can be confirmed retrospectively to determine if there are differences in the often self-reported gender and sex in publicly available sample sets.

The influence of rare genetic variants on RNA is another poorly explored area of physiology. Rare diseases have vastly enhanced our knowledge of phenotypic diversity, yet how these variants alter gene expression, splicing, and noncoding RNA remains underexplored. Rare disease patient-centric RNA sequencing could elucidate many genotype-phenotype associations but is challenged by the obtainable tissue. As patients with rare diseases are spread across the globe, the process for obtaining biospecimens, especially for pediatric patients, can vary widely. Some patients may not have access to clinical laboratories that can collect, process, and store samples needed for RNA sequencing. Growth of induced pluripotent stem cells (iPSCs) from these patients may enable broader development insights when paired with RNA sequencing, but these initiatives require more investments and biobanking initiatives. In addition, the complex treatment dynamics of gene therapies for these patients would be paired well with RNA sequencing explorations for immune alterations and outcome biomarkers (128).

RNA sequencing does provide several ethical challenges that need consideration. The extraction of variants within RNA sequencing data is associated with several issues. The analysis of variants from existing data needs to be performed carefully, as not all patients consented to variant insights and the risks that can be associated with them. From disease risk variants to population structure, many patients were not informed of the risks these analyses may entail. It remains uncertain how many variants within each RNA sequencing data set may potentially reidentify a sample, especially when combined with deposited metadata. As IRB offices and projects continue to attempt linkage of RNA sequencing to metadata of samples, it also continues to complicate the potential for reidentification of any specimen, where these risks need to be carefully considered in the study design and consenting process. Although science has advanced significantly by increasing physiological insights linked to each sample, patient protection must be accounted for in already generated data and future experiments. Given the more challenging nature of maintaining data under the protection of dbGaP or locally maintained repositories (such as REDCap), this challenge should never be underestimated when moving into human RNA sequencing.

## USAGE OF RNA SEQUENCING

The growth of RNA sequencing has shown promise in revolutionizing precision medicine and other applications in physiology. To conclude this review, we aim to highlight several of our first-hand experiences with new directions for RNA sequencing.

### Multidimensional Deconvolution of RNA

Our team has been working to develop and implement precision transcriptomics. Building on our experiences in whole genome sequencing rare diseases, we began exploring what can be found in RNA sequencing human cohorts that might contribute to disease insights. Our first explorations were centered within critical care units of the hospital, especially the pediatric or neonatal intensive care unit (PICU/NICU). Our work utilized a blood collection system, PAXgene, that facilitated the easier clinical collection and stabilization of all RNA within the blood specimens. This was processed with paired-end total RNA sequencing (ribosomal and globin reduced) on the Illumina short-read platforms.

**Figure 7.**
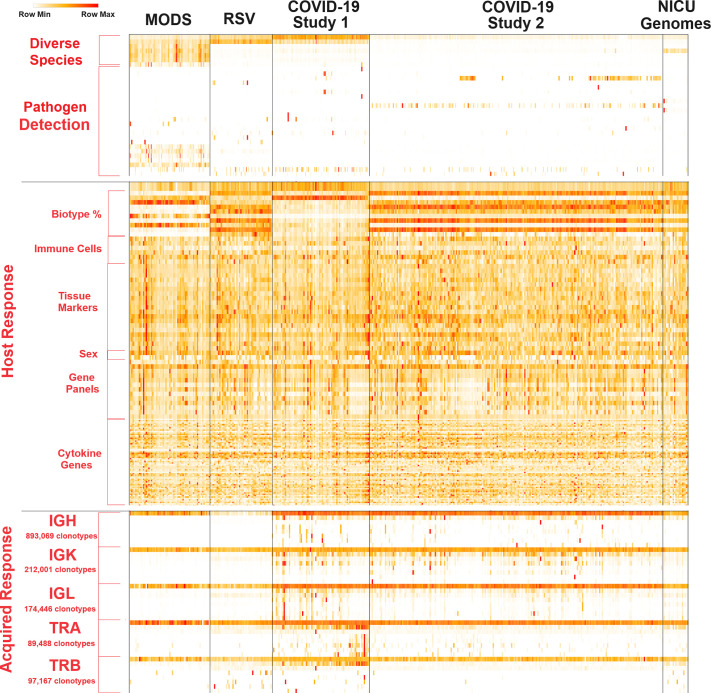
Corewell health internal patient RNA sequencing mapped for multidimensional insights from PAXgene tubes in human ribosomal reduced short-read, paired-end RNA sequencing. The heatmap is shown as a *z*-score colored per row, with the lowest in white and the highest in red.

Three projects/cohorts were started as case versus control statistical designs but morphed into precision discoveries out of exploring the complex heterogeneous data of clinical samples. Two additional studies were then completed for more precision transcriptomic insights to make five cohorts of RNA sequencing (Fig. 7). Figure 7 shows the multiple areas of mapping knowledge that include diverse species, host gene responses, and the immune repertoire based on acquired immune response. Throughout these five studies of analysis, our team identified tools to elevate the mapping of RNA reads to enhance discovery, where we settled on a set of tools able to identify foreign RNA (295), human transcript alignments (62), and the complex immune repertoire (296). As Fig. 7 shows, these studies produced batch effects in their data mapping but provided statistical power within each cohort to make discoveries for individual samples.

Our first study was that of PICU patients with multiple organ dysfunction syndrome (MODS) paired with sedation controls matched to age and sex. The MODS phenotype, defined as two or more organs in failure, proved too heterogeneous for routine statistical mapping tools to resolve significance relative to controls (297). However, a subset of the MODS patients required extracorporeal membrane oxygenation, where statistics were powered to identify risk expression profiles associated with various histone genes and neutrophil extracellular trap (NETosis) production (298).

Our second study of patient samples focused on the NICU/PICU to address respiratory syncytial virus (RSV) relative to sedation controls in young children (299). Within the RNA sequencing data, we found that patients with RSV had significantly elevated immune system genes, including interferon and cytokine signaling. The expression of several sex chromosome genes was also significantly altered in RSV, suggesting reasons why males tend to have higher rates of hospitalization in children.

The third blood study addressed adult hospitalizations early in COVID-19 relative to healthy adults without COVID-19 (300). The study included 11 cases that resulted in death, with 25 COVID-19 patients who survived. It was performed before any clinical trials were started on COVID-19 vaccines. The statistical analysis relative to controls elucidated increased gene signatures of neutrophil mechanisms, secretory granules, and NETosis with decreased signatures of lymphocytes. However, when mapping the data for each patient to those genes and pathways and performing unsupervised sample clustering, it was also realized that two subclusters within the patients suggested different activations or different temporal states. This highlights one of our team’s most valuable lessons in clinical RNA sequencing utility: traditional statistical models are often built on binary comparisons and outcomes. Disease mechanisms can be heterogeneous and require more than one answer. It is always suggested that mapping data from significance be studied within individual samples to explore their heterogeneity, for example, by performing unsupervised clustering. This suggests that even in COVID-19, understanding the immune response could hold potential for stratifying clinical trials based on the immune states (301).

### Toward Precision, Patient-Level Transcriptomics

All three of the above studies showed heterogeneous individuals, where we realized the power of studying heterogeneous biology within RNA sequencing. As the heatmap of Fig. 7 shows, within many of our cohorts, there are unique patients with strong red bands in nearly every category of data mapping, suggesting that patients can have unique elevations. The real challenge is understanding what signatures are real biological insights relative to the background noise of the data. Instead of performing case versus control statistical testing, we refocused our statistical approaches to outlier mapping. We used Z-score outliers of human genes per patient, matching the genes to enriched gene ontology terms to identify patient-altered biological pathways. These pathways were then compared with the additional mapping data, such as non-human species and the antibody or T-cell receptors of the immune repertoire. As with rare disease whole genome sequencing (9), our team hypothesizes that linking biological heterogeneity through this type of precision transcriptomics may guide better future diagnoses and treatment plans for each patient.

We first showed the value of understanding infection mapping using RNA sequencing within the MODS cohort (297). Patients with MODS had three time points of blood collection to represent early care to the recovery period. RNA sequencing data analysis was blinded to any clinical data to avoid bias in the detection of signals. Our team showed several patients within the cohort with outlier detections of *Streptococcus* species, all later confirmed to have clinically detected strep infections. The RNA sequencing tools provided subspecies of insights, whereas the clinical data confirmed a broad Strep infection. Within one of the patients, we showed a later blood sample to have outlier levels of *Staphylococcus aureus*, where later in the clinical data correlations, we confirmed a hospital-acquired staph infection was present in that patient.

One of the unique mappings in a patient with MODS was read to *Morbillivirus*, a known cause of measles, which was present at two time points in that patient. This patient presented none of the symptoms of measles; thus, the signal was thought to be noise in RNA sequencing. Yet every other sample processed in the MODS and the other cohorts had zero reads aligned to *Morbillivirus*, suggesting this was not noise of the technique. From the RNA reads aligned to *Morbillivirus*, we further extracted the reads, reassembled them into a construct at the sequence level, and a BLAST search of the base level sequence identified high homology to the measles vaccine. In the clinical note follow-up due to the read detection, it was confirmed that the patient received a measles vaccine a few days before hospitalization with a bacterial infection.

**Figure 8.**
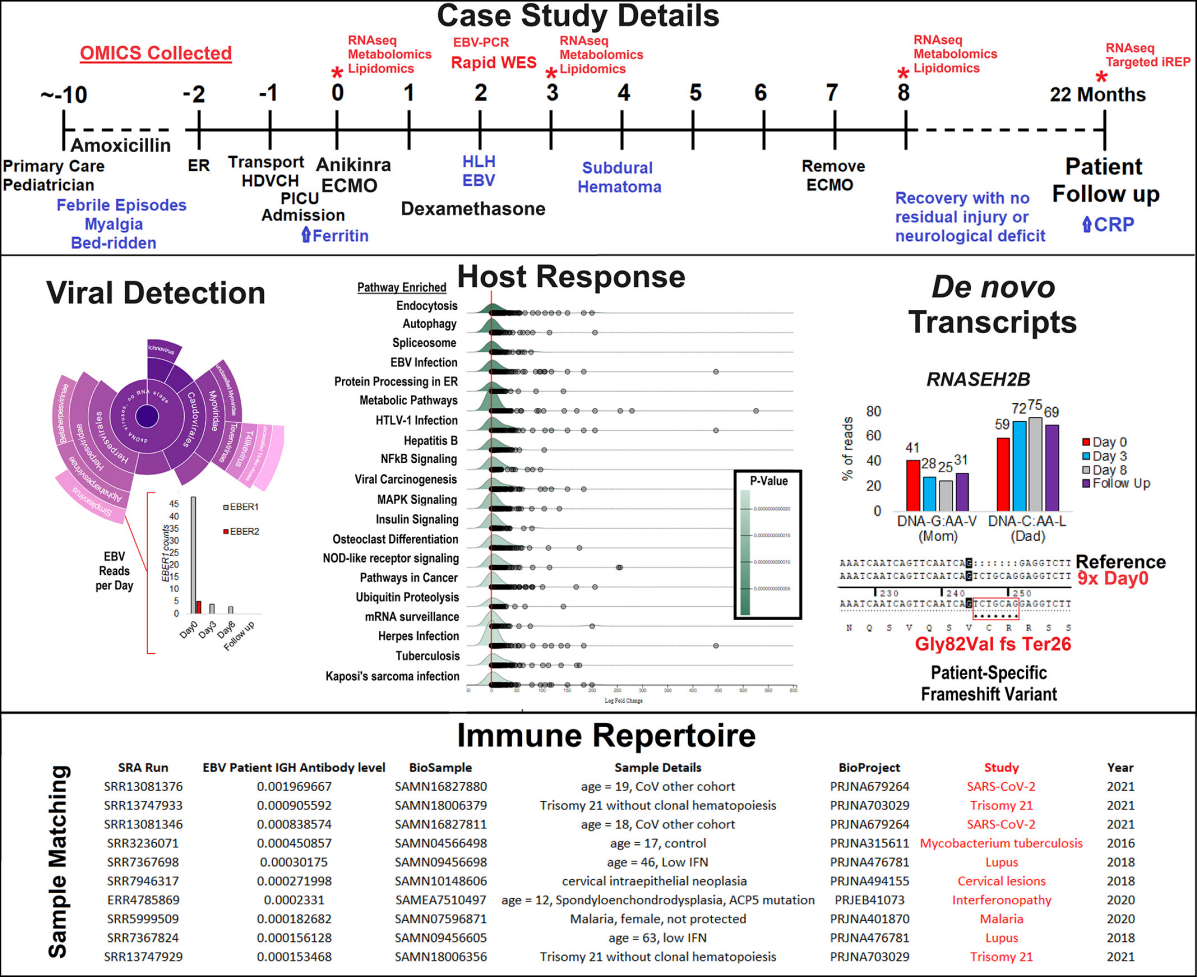
A case study of multiple organ dysfunction that highlights the role of environmental regulation of nonsense-mediated decay transcripts and physiological outcomes. *Top*: shows the clinical course with omic datasets collected in red. *Middle*: shows the patient mapping data from viral detection of EBV, outlier gene ontology enrichment terms relative to the larger cohort of patients, and the allelic imbalance of the RNASEH2B gene as determined by RNA sequencing. *Bottom*: shows the antibody sequences extracted from RNA sequencing matched to thousands of samples within the NCBI SRA. EBV, Epstein–Barr virus; NCBI SRA, National Center for Biotechnology Information Sequence Read Archive. *Time points of omics collection.

This work highlights the potential of bacterial and viral detections of RNA sequencing that can be combined with other mapped data from RNA sequencing to revolutionize our knowledge of the complex physiology of infections. To highlight the potential of heterogeneous physiological responses within individuals, we highlight one of the patients with MODS, where we discovered a process we coined virally induced genetics (Fig. 8). The patient, in their teens, presented symptoms of an infection that progressed into MODS that included hemophagocytic lymphohistiocytosis (HLH) due to a clinically confirmed Epstein–Barr virus (EBV), which was detectable at in the RNA sequencing data. The patient’s signatures showed unique outlier genes connected to EBV response and signs of altered RNA processes such as RNA surveillance and NMD.

A rapid clinical exome of the patient found variants for a possible autosomal recessive rare disease in the *RNASEH2B* gene, but the clinical presentation did not match that rare disease. One of the variants they inherited in *RNASEH2B* from their dad was predicted to be benign. The variant they inherited from their mom was a splice change with unknown transcript and protein outcomes. Making sense of a splice variant outcome on a protein requires tools such as RNA sequencing to determine the new splice change that occurs. The patient’s RNA sequencing confirmed the presence of a new splice that caused a frameshift and early truncation of the RNASEH2B protein. However, using the variants, we could also track in the RNA sequencing the splice variant (maternally inherited) usage was highest at the height of their EBV infection, with recovery showing a higher use of their *RNASEH2B* gene inherited from dad when they recovered. This suggested that NMD could regulate the splice variant and early stop codon frameshift but that something altered NMD during the EBV infection. This aligns with observations that EBV can suppress NMD to replicate, which we suspect resulted in a rare autosomal dominant form of the disease phenotype in the patient only during their viral infection of the blood cells (275).

Further processing the immune repertoire, we could find an antibody signature elevated later in the patient’s RNA sequencing. There was suspicion that the antibody signature could potentially be a risk factor for autoimmune disease based on the patient’s transient rare disease, HLH, and NETosis. What makes the immune repertoire so powerful, over something like antibody serological insights, is the ability to get sequence-level acquired response data that can be matched to other RNA sequencing samples. With the patient’s antibody profile, we could search for blood PAXgene data from the NCBI SRA (283) to find similar datasets that link to other disease phenotypes. Nearly all of the matched samples were from infection patients or those with autoimmunity such as lupus, further suggesting the patient may be at risk for autoimmunity. A 2-year follow-up of the patient showed many signs of these autoimmune issues that were being treated clinically following their PICU EBV complication. This ability to link acute infections to long-term disease risks is one of the most promising aspects of precision transcriptomics.

As the patient in Fig. 8 shows, COVID-19 and the SARS-CoV-2 infection seem to suggest shared responses within antibodies of the immune repertoire. Our COVID-19 study also showed through precision transcriptomics that SARS-CoV-2 is a potent suppressor of immunity in many patients, which can result in the reactivation of dormant viruses such as EBV, shingles, or Torque teno virus, as observed through outlier mapping (300). Other patients had bacterial signatures as outliers for species such as *Pseudomonas*, which are normal flora that can be elevated in immune-suppressed individuals. This suggests that COVID-19, through immune suppression, can elevate patient-specific clinical secondary infections, so traditional cohort-level mapping approaches would not be significantly powered to measure individual species. Our team has rapidly been expanding these precision transcriptomic tools into other clinical areas, such as inborn errors of immunity, pregnancy complications, rare diseases, traumatic brain injury, and gene therapy, which have the potential for complex interactions of genetics, immune system dynamics, and infections on medical and physiological questions.

Not only can these tools be applied to new sample collections, but they can also be applied to older data within the NCBI SRA. For example, using multidimensional mapping tools, we processed 8,274 RNAseq samples from 132 different experiments for skin samples (302). These tools elucidated many novel discoveries in microbiome-host dynamics, such as the presence of *Bacillus megaterium* correlated with antibody production in patients with hidradenitis suppurativa. A few powerful computers could synergize skin data into sex differences, antibody profiles across data, novel viruses in humans (equine infectious anemia virus), and elucidate the methods that produce the best mapping of specific data that might be desirable to future studies. This work was led by a medical student passionate about dermatology, fitting the computational analysis during the rigorous medical school training. The potential to pair these mapping tools with artificial intelligence (AI) and the broader resources of the NCBI SRA holds incredible promise to reach a new era of precision medicine.

### Integration with Other Omics

RNAseq has revolutionized transcriptomics by enabling high-resolution analysis of gene expression, splicing events, and noncoding RNA profiles (303). However, the complexity of biological systems often necessitates a multiomics approach to capture the full spectrum of molecular interactions (304). Integrating RNAseq with other omics layers, such as genomics, epigenomics, proteomics, metabolomics, and spatial omics, provides a more comprehensive understanding of cellular physiology, disease mechanisms, and therapeutic targets (305). For instance, the combination of RNAseq with whole genome or exome sequencing enables linking genomic variants, such as single-nucleotide polymorphisms (SNPs) and indels, to gene expression profiles, facilitating the identification of eQTLs, sQTLs, allele-specific expression in disease pathogenesis, and the impact of rare genetic variants on transcriptome regulation (251). As is currently being unraveled in the case of cancer (306), that >90% of cancers are initiated by environmental exposures (exposome), leading to genetic changes in the subsequent proliferating cancer cells. In response to the altered cancerous cell, cancer-specific metabolism, which is genetically reprogrammed, facilitates and sustains this new cancerous cellular growth. These three “omics” work together to create a new hostile environment for the host. Current science is unraveling these complex interactions in an integrated and holistic approach, which gives new hope for prevention and treatment strategies.

Similarly, integrating RNAseq with epigenomics, which focuses on DNA methylation, histone modifications, and chromatin accessibility, provides insights into transcriptional regulation when combined with techniques like ATAC-seq, bisulfite sequencing, ChIP-seq, and nanopore direct sequencing (307–309). This approach allows researchers to identify regulatory elements controlling gene expression, link epigenetic modifications to differential expression and splicing, and explore gene regulation in developmental and disease contexts (308, 310).

Further integration of RNAseq with proteomics addresses the imperfect correlation between transcriptome data and protein abundance due to posttranscriptional regulation (311, 312). By combining transcriptomics with proteomics techniques such as mass spectrometry and protein arrays, researchers can link gene expression to protein abundance and activity, uncover translational control mechanisms and posttranslational modifications, and study protein-protein interaction networks influenced by transcriptomic changes (313, 314). Similarly, pairing RNAseq with metabolomics, which profiles small molecules and metabolites via methods like NMR, UPLC-MS, and GC-MS (315), highlights gene-to-phenotype relationships and uncovers metabolic pathways regulated at the transcriptional level (316, 317). This integration is particularly valuable for understanding metabolic flux (318), disease biomarker discovery, and exploring the molecular underpinnings of conditions such as cancer, neurodegeneration, and metabolic disorders (318, 319).

In addition, spatial omics, including spatial transcriptomics and proteomics, merge RNA expression data with spatial context to map cell type-specific gene expression within tissues, study tumor microenvironments, and identify spatial coexpression patterns in developmental biology (320). Single-cell omics further enhances RNAseq by combining it with single-cell ATAC-seq, proteomics, or spatial data, allowing deep characterization of cellular heterogeneity, elucidation of developmental trajectories, and mapping of cellular ecosystems in cancer, immune responses, and tissue regeneration (185, 321, 322).

Despite these advances, multiomics data integration presents challenges, including harmonization across platforms with differing resolution and scale, handling batch effects and variability, and interpreting biological relevance within complex datasets (323, 324). Additional practical challenges for the multiomics community include data acquisition, integration (machine learning, unsupervised, supervised, deep learning), analysis, and storage, which require specialized equipment, trained personnel, and abundant financial resources (325, 326). These factors impede the practical and timely clinical translation of this work to the bedside. Additional challenges include a lack of standardization of sample collection, missing data and samples, batch effects, and data complexity, to mention a few. However, some of these barriers are being explored and overcome in the wearable device arena, which offers real-time, continuous treatment and environmental exposure monitoring (327).

### Temporal-Rhythmic Expression Dynamics

Circadian rhythms are critical for maintaining physiological homeostasis, where much of our knowledge of circadian rhythms comes from a balance of transcriptomics and protein assessments. This section highlights the power of following techniques like transcriptomics over temporal analyses. Every tissue and cell in the body has a circadian rhythm that will respond to Zeitgebers or time givers. Zeitgebers include light, food intake, and physical activity (328). The suprachiasmatic nucleus (SCN) in the hypothalamus of the brain responds to light cues from the retina, entraining or driving the central circadian clock as well as regulation of body temperature (329), melatonin secretion (330), and signaling to peripheral tissue clocks through autonomic innervation (331). Many studies have shown that aberrant light exposure leads to negative health consequences due to shifts in the circadian phase and difficulty entraining the circadian rhythm (332–334). Multiple recent studies indicated that night-shift workers are at an increased risk of cardiovascular disease due to circadian disruption (335–337). Due to light-induced circadian disruption, many physiological consequences arise, including sleep/wake cycle misalignment and metabolic dysfunction (333, 334, 338).

Peripheral clocks are complex, as each tissue has its clock and will respond differently to different Zeitgebers. The liver and adipose tissue rely on food intake cues for normal tissue rhythmicity (339–342). Mistimed eating can lead to metabolic dysfunction or glucose intolerance (343, 344), increasing the likelihood of obesity and its associated comorbidities (345, 346). The kidney and vasculature also benefit from adequately timed eating, as demonstrated by interventions that alter the timing of feeding (347–349). The Pollock group (coauthors of paper) found that mistimed feeding of healthy food in mice (food available during the light/inactive phase only) completely reversed the blood pressure rhythms, with blood pressure peaks during the light phase and dips during the dark phase, as well as increasing aortic stiffness. Interestingly, these mice maintained a normal diurnal rhythm of sodium excretion (350). Sutton et al. (351) showed that prediabetic obese men on a 6-h time-restricted feeding (food intake from 8:00 AM to 2:00 PM) intervention had significantly reduced blood pressure compared with controls with a 12–15 h feeding window. The Pollock laboratory has also recently shown that time-restricted feeding intervention in an animal model of diet-induced obesity significantly reduced renal fibrosis and aortic stiffness, highlighting the importance of timing food intake for cardiorenal function (352). Exercise affects circadian rhythms by altering the phase of melatonin to improve the sleep/wake cycle (353, 354) and diurnal fluctuations of muscle strength (355). Exercise has also been found to improve blood pressure rhythms with increased dipping at night (356). These reports further highlight a connection between maintaining circadian rhythms through food cues and/or physical activity, critical to understanding whole body physiology.

RNA transcription is also circadian-dependent (357, 358). Rhythmic RNA expression can occur in up to 80% of the total RNA transcripts in specific tissues (359–361). Menet et al. (357) elegantly showed rhythmic gene expression patterns in nascent RNA and mRNA throughout a 24-h period. Studies have also shown that the recruitment of RNA polymerase II to promoter sites occurs on a diurnal rhythm, along with histone methylation, H3K4me3, and H3K36me3, which are associated with the transcription start site and gene splicing (362). RNA transcription in eukaryotes requires DNA-dependent RNA polymerases that, in turn, utilize transcription factors binding at promoter regions to enhance the assembly of (or repress) the initiation complex (363, 364). Thus, small changes in transcription factor expression (365) and/or nuclear localization (366) can result in dramatic changes in RNA expression (365) and cellular fate (366) by regulating the assembly and transcriptional capacity of the holoenzyme. RNA sequencing studies with application to physiology are susceptible to the time at which the samples were initially obtained, and even 2-h sample-acquisition differences between groups can result in the false attribution of differentially expressed genes to treatment rather than properly to time-of-day (359, 360).

About 30% of all mammalian genes have oscillating expression over a 24-h period. These rhythmic genes regulate the circadian transcription-translational feedback loop, such as *Arntl* (aka *Bmal1*), *Clock*, *Per1*, *Per2*, *Cry1* and *Cry2*, and many non-circadian-associated genes (367–369). The central molecular clock proteins, Bmal1 and Clock, are transcription factors (370, 371) that bind E-Box DNA motifs to drive the expression of thousands of genes either indirectly by increasing chromatin accessibility for the binding of other transcription factors or directly by recruiting RNA polymerase (358, 372–374). Bmal1 is sensitive to regulation by other proteins (370, 371) and pharmacological interventions (375–377) to drive a phase-shift (delaying or accelerating) in *Bmal1* expression. It is then conceivable that differentially expressed genes identified from RNA-sequencing studies may amount to phase shifts in *Bmal1* or other circadian-relevant transcription factor expression. Thus, RNA-sequencing studies must report the time and conditions at which samples were obtained, and for increased scientific rigor, more than one time-of-day endpoint may need to be incorporated.

There are still many questions about why and how the circadian regulation of specific genes affects physiological function. Of note, the capacity of CLOCK:BMAL1 to bind to E-Box motifs is moderated by tissue and cell-specific chromatin accessibility (373) and local DNA-shape and histone modifications (372), indicating clock protein regulation of rhythmic RNA expression is distinct to each tissue. Tissue-specific and rhythmic regulation of chromatin accessibility results in a transcriptional landscape that is unique and variable for each cell type based on time of day, potentially leading to time-sensitive and clinically relevant tissue-specific responses to intervention and/or challenge, whether by pharmacological intervention or some other insult (360). For example, we know that circadian expression of genes in the kidney plays a critical role in electrolyte homeostasis (378), and dysregulation of these renal transcriptomic rhythms contributes to a blunted capacity to respond to environmental changes and increased renal injury upon hypertensive challenge (378–380). Circadian regulation in the liver controls lipid metabolism (381), detoxification (382), and nutrient uptake (383). Studies in rodents and humans show that exercise affects the rhythmicity of core circadian genes and genes associated with stress, like *Herpud1* (384, 385), expressed explicitly in the skeletal muscle. Microarray analysis of the left ventricle of the heart 8 h post-induction of myocardial infarction indicated significant differences in the inflammatory response, metabolic pathways, and transcription regulation based on whether the infarct was initiated during the beginning of the mouse inactive (lights-on) or active (lights-off) period (386). A similar time-of-day sensitive difference in RNA transcription has also been observed in the human immunologic response to hypoxia (387) and in the responsiveness of various cancers to chemotherapeutics (388, 389).

These observations suggest studies interrogating the transcriptomic response to perturbation may be sensitive to the time the stressor or challenge began. Therefore, considering not just the time at which the sample was obtained but also the time at which the challenge was initiated may prove critical for future RNA sequencing studies to improve pathway identification. As the access and utilization of RNA sequencing technology expand in physiology, physiologically relevant parameters, such as when the experimental intervention took place and when the sample was obtained, will become increasingly critical for precise pathway analysis and for enabling a systematic and consistent comparison between published studies. These metadata are rarely present in previous RNA sequencing experiments, and this represents one of the most likely factors for heterogeneous insights within experiments, especially as the tools are applied to precision medicine approaches.

### Direct RNA Sequencing

One of the most unique up-and-coming RNA sequencing platforms is the nanopore direct method, which removes the need for cDNA generation by directly sequencing the RNA molecules through protein pores. Our team has applied this approach to two laboratory-controlled cell culture experiments, highlighting the potential of the tools for exploring the signaling dynamics of RNA base modifications in brain and kidney physiology.

**Figure 9.**
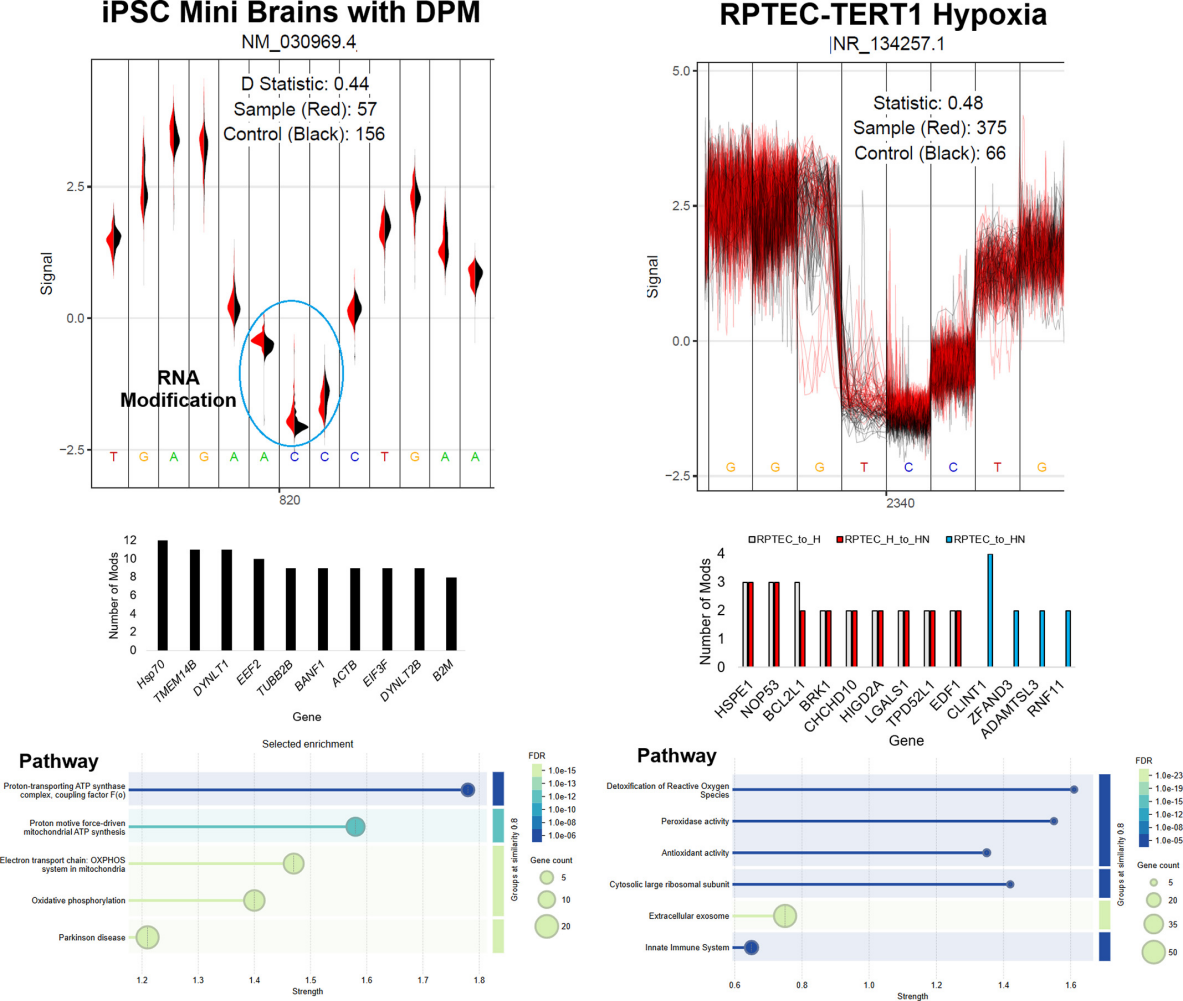
Nanopore-based direct RNAseq elucidates RNA modification signal transduction due to environmental alterations. *Left:* shows data from iPSC-derived mini brains exposed to diesel particulate matter (DPM). *Right:* show data from RPTEC-TERT1 kidney cells exposed to 1% hypoxia. *Top*: show representative voltage plots used to map modification sites with the mini-brain shown with violin representation and the kidney plot shown as raw voltage overlay plots. *Middle*: show top genes with the number of identified modifications within the transcripts. The kidney panel shows the differential modification detections over the three labeled experiments. *Bottom*: show pathways enriched for modified genes based on STRING analysis. H, hypoxic exposure; HN, hypoxic to normoxic exposures; iPSC, induced pluripotent stem cell.

Using induced pluripotent stem cell (iPSC) derived mini-brains, we combined short-read RNA sequencing, single-cell RNA sequencing, and nanopore direct RNA sequencing for the role of diesel particulate matter (DPM) exposure on human brain development (390). Short-read RNA sequencing showed that gene expression within the mini-brains was altered with DPM treatment, with the more expensive single-cell RNA sequencing then showing that the min-brains had 19 cell types with significant gene expression changes in several cell clusters. Genes altered in expression were associated with oxidative phosphorylation. The nanopore direct RNA sequencing was able to compare the raw voltage data of control or DPM exposure mini-brains to detect subtle shifts that are indicative of RNA base modifications (Fig. 9, *left top*). Interestingly, many of the genes where a modification occurred showed multiple sites within the transcript to be modified (Fig. 9, *left middle*). The genes where these modifications occurred were not random but showed changes to RNA transcripts significantly enriched to mitochondrial function and oxidative phosphorylation (Fig. 9, *left bottom*).

A second study linked hypoxia (1% oxygen) exposure within confluent (nonproliferative), sheer-stress-treated (391) human renal proximal tubule epithelial cells (RPTEC) to signal biology (122). Using short-read RNA sequencing, we established a sheer stress model that, with additional immunofluorescence, showed how hypoxia alters cilia physiology within a cell culture model (391). RNA sequencing also established that this model can capture a transient state of epithelial cells for epithelial-mesenchymal transition, similar to how hypoxia can induce cancer metastasis in epithelial cells.

Using this RPTEC hypoxia model, we applied direct nanopore RNA sequencing, which identified RNA modification sites (Fig. 9, *right top*) (122). We used three cell groups and performed three comparisons of modifications for this study: *1*) control cells versus 48 h of hypoxia exposure (RPTECT_to_H), *2*) 48 h of hypoxia exposure versus cells exposed for 48 h hypoxia followed by 48 h with normal oxygen (RPTEC_H_to_HN), and *3*) control to the reoxygenated cells (RPTEC_to_HN). This work also suggested that RNA base modifications occurred multiple times within multiple transcripts (Fig. 9, *right middle*). Interestingly, the reoxygenation of the cells for 48 h seemed to reverse most modification sites, with some modifications that were stable for longer, which we hypothesize is based on RNA half-life differences. The genes that showed modification due to hypoxia were not random but enriched for pathways such as reactive oxygen species and innate immunity, both well established in hypoxia response. One of the top modified transcripts was *RHOA*, a well-established control gene for cancer metastasis (122).

Having two models where case versus control direct nanopore-based statistics suggest targeted modifications to genes involved in physiological pathways already established to logical outcomes of the models suggests that RNA base modifications may have functions in signal transductions. This could be similar to protein phosphorylation, which revolutionized our insights into signal transduction (392, 393). RNA base modifications are well established to control RNA transcript turnover and stability (227). This could have rapid signal potential through RNA to protein translation rates that could take environmental signals and metabolic control of enzymes to manifest amplification to cellular physiology. If true, direct RNA sequencing with nanopore could also revolutionize our signal transduction knowledge over the coming years if applied to broad physiological experiments, especially those where control and treatment can be compared.

### Artificial Intelligence and Machine Learning

As the dimensions of data from RNA sequencing increase to include splicing dynamics, RNA biotype insights, foreign RNA (such as bacteria and virus), the immune repertoire, cell-specificity, tissue spatial dynamics, cyclical expression dynamics, RNA modifications, disease-centered profiles, sex/gender differences, and the many other aspects of RNA biology, the ability to understand the complex system exceeds what scientists can comprehend. There is a growing need for machine learning and artificial intelligence tools to help organize and interpret the complex biology within RNA. Early in this phase, the data integrations and models often focused on single factors such as expression profiles (394), immunotherapies (395), single-cell atlases (270, 396, 397), and spatial transcriptome deconvolution (398–400). One of the exciting emerging areas of RNA and artificial intelligence is the rapid growth of RNA structure predictions that have long challenged our knowledge of how RNA functions within a cell (401, 402). As discussed throughout this review, if we imagine an AI system that can inform physiology around homeostasis and heterogeneity, there must be modeling that incorporates the many dimensions of RNA knowledge addressed throughout this review.

### Interventions with RNA

As our knowledge of RNA has expanded, it has opened the door for therapeutic interventions. Delivery of RNA and RNA-regulating molecules is rapidly emerging therapeutically to manipulate gene expression (403), targeting established genes in infectious diseases, cancers, the immune system, and Mendelian rare diseases (128). This means that the scientific results of short-read, long-read, single-cell, and spatial RNA sequencing detection of RNA involved in disease transformation can be targeted with synthesized mRNA (to elevate), antisense RNA (to reduce), or CRISPR-mediated expression regulation (elevate or reduce). Multiple of these molecules are now FDA-approved and have direct clinical usage, highlighting the future of RNA-based therapies (128).

RNA modifications, including methylation, an epigenetic modification that is very different from the mechanisms that govern DNA methylation, have an impact on the half-life of therapeutic RNA. Protein regulation of the RNA methylation includes “writers,” “erasers,” and “readers” that respectively deposit, remove, and recognize methylated RNA. RNA methylation at many levels, particularly N6-methyladenosine (m6A), 5-methylcytosine (m5C), N3-methylcytosine (m3C), N1-methyladenosine (m1A), and N7-methylguanosine (m7G), has been suggested as disease therapeutic targets (404). More work is needed in this rapidly emerging area on how best to control synthesized RNA therapies.

Delivery of the RNA molecules can be based on viral packaging or lipid-based particles with some target specificity. The specificity can be further selected using the expression construct coded within the delivery system based on cell-specific promoters/enhancers driven by extensive single-cell level expression integrations (405). Many of the current clinical trials of gene therapies were based on bulk tissue-specific RNA expression datasets, where we anticipate a new era of cell-level expression control of gene therapy systems to reduce off-target profiles and complications (128, 406). Spatial transcriptomics applied to disease will yield single-cell, region-specific insights that allow even further refining of disease-specific expression control (407). One of the most significant advancements in RNA, which elevated to the Nobel Prize, was the understanding of how RNA modifications contribute to the half-life of RNA (408–410), which elevated the ability to develop mRNA-based vaccines during COVID-19 (411).

Developing drug targets needs relative abundance and knowledge of the function of RNA transcripts paired with knowledge of protein abundance and outcomes, emphasizing expression dynamics like circadian rhythms that are often overlooked. These systems are not always consistent between humans and model organisms such as rodents, thus requiring more human systems for developing RNA-centered therapies. The further development of human wet laboratory models, such as induced pluripotent stem cells (iPSCs) and other ex vitro models, holds promise in validating RNA therapies before human clinical trials. These human-centered RNA models could enable the expansion of heterogeneity insights that often lead to failed clinical trials and expanding lists of adverse outcomes following FDA approvals (128). It is also critical to elevate programs in biomarker assessments for RNA gene therapies even after they have been FDA-approved, using the RNA sequencing tools addressed throughout this article. This is critical given the often low sample size of clinical trials that cannot capture the broad heterogeneous responses within cells to the RNA and the immune response to the packaging material (128). The advancing AI tools that integrate the broader RNA insights are likely to inform more on the heterogeneous risks of RNA therapies and to develop more homeostasis controls that optimize the “Goldilocks” needs of physiological control without adverse outcomes.

The introduction of machine learning and artificial intelligence to large volumes and diverse types of RNA data can guide many processes of RNA-based therapies from delivery, cell-specific expression, disease-centric control, and fine-tuning the system to identify the Goldilocks of expression to have just the right amount of RNA function for physiological needs while accounting for cyclical dynamics and feedback loops. As data increases and computational tools are developed to integrate better RNA sequencing platforms mentioned earlier, our ability to develop a toolbox of resources in gene-based therapies grows. This toolbox of RNA can likely enable lower-cost, faster-moving therapy opportunities that reflect the alignment of physiological heterogeneity and homeostasis that define precision medicine in the future.

## CONCLUSIONS

RNA sequencing continues to expand in usage and methods. The next decade will likely see new technologies and techniques for processing RNA, with significant advancements in cell physiology mechanisms. The emerging market competition in sequencing is likely to drive innovation and cost reductions that will facilitate more RNA sequencing into physiology projects. The tools will be applied to broad physiological systems and model organisms, while their use for processing clinical samples will also elevate. Our knowledge of disease will be enhanced as the tools are applied. The challenges of selecting which tool to use for which samples will not resolve soon, with the trade-off for depth of knowledge relative to costs getting more difficult as costs decrease on platforms such as single-cell and spatial. The depth of data generated will also get more challenging, requiring laboratories to understand existing data to inform new experimental designs. Like the roots of all physiology, observational discoveries will be plentiful for laboratories utilizing RNA sequencing, better designing hypotheses built around the massive volumes of RNA insights, and the depth of diverse samples already available. More importantly, every physiologist will now be powered with tools that can better resolve the complexities of homeostasis and heterogeneity of disease, enabling the future of precision medicine that was promised with the completion of the human genome.
